# Strategic Amyotrophic Lateral Sclerosis Australia–Systems Genomics Consortium (SALSA-SGC): cohort profile

**DOI:** 10.1136/bmjopen-2025-110906

**Published:** 2026-06-18

**Authors:** Anjali K Henders, Laura Ziser, Fleur C Garton, Lorel Adams, Karalyn Ernst, Sarah Furlong, Judith Anne Heads, Susan Heggie, Ruth Krasniqi, Madhura Bhadravathi Lokeshappa, Srestha Mazumder, Elyshia McNamara, Amanda MacShane, Linda Mekhael, Lorelle Nunn, Bronwen Orden, Julie Ryder, Kathryn Thorpe, Marie Toubia, Leanne M Wallace, Dina Wickremeratne, Emma Windebank, Beben Benyamin, Shyuan Ngo, Garth Nicholson, Roger Pamphlett, Frederik J Steyn, Peter M Visscher, Kelly L Willams, Robert Henderson, Matthew C Kiernan, Nigel Laing, Susan Mathers, Pamela A McCombe, Merrilee Needham, Dominic Rowe, David Schultz, Paul Talman, Steve Vucic, Ian P Blair, Allan F McRae, Naomi R Wray

**Affiliations:** 1 Institute for Molecular Bioscience, The University of Queensland, Brisbane, Queensland, Australia; 2 Motor Neuron Disease Research Centre, Macquarie University, Sydney, New South Wales, Australia; 3 Department of Neurology, Flinders Medical Centre, Bedford Park, South Australia, Australia; 4 Department of Neurology, Royal Brisbane and Women’s Hospital, Herston, Queensland, Australia; 5 Neurology, Calvary Health Care Bethlehem, Caulfield South, Victoria, Australia; 6, Neuroscience Research Australia, Randwick, New South Wales, Australia; 7 Harry Perkins Institute of Medical Research, Perth, Western Australia, Australia; 8 Australia Brain and Nerve Research Center, Concord Hospital, Sydney, New South Wales, Australia; 9 Australian Institute for Bioengineering and Nanotechnology, The University of Queensland, Brisbane, Queensland, Australia; 10 Australian Centre for Precision Health, University of South Australia, Adelaide, South Australia, Australia; 11 School of Biomedical Sciences, The University of Queensland, Saint Lucia, Queensland, Australia; 12 Concord Clinical School, Concord Hospital, Sydney, New South Wales, Australia; 13 Sydney Medical School, The University of Sydney, New South Wales, Sydney, New South Wales, Australia; 14 Pathology, The University of Sydney, Sydney, New South Wales, Australia; 15 Brain and Mind Centre, The University of Sydney, Sydney, New South Wales, Australia; 16 Department of Neuropathology, Royal Prince Alfred Hospital, Sydney, New South Wales, Australia; 17 Nuffield Department of Population Health, University of Oxford, Oxford, UK; 18 University Of Queensland Centre For Clinical Research, Herston, Queensland, Australia; 19 Department of Neurology, Royal Prince Alfred Hospital, Sydney, New South Wales, Australia; 20 Neuroscience, The University of New South Wales, Sydney, New South Wales, Australia; 21 UQ Centre for Clinical Research, University of Queensland, Brisbane, Queensland, Australia; 22 Department of Neurology, Fiona Stanley Hospital, Perth, Western Australia, Australia; 23 CMMIT, Murdoch University, Murdoch, Western Australia, Australia; 24 School of Medicine, University of Notre Dame, Perth, Western Australia, Australia; 25 University Hospital, Deakin University Faculty of Science and Technology, Geelong, Victoria, Australia; 26 Concord Clinical School, Concord Hospital, Concord, New South Wales, Australia; 27 Department of Psychiatry, University of Oxford, Oxford, UK

**Keywords:** GENETICS, Longitudinal studies, Motor neurone disease

## Abstract

**Abstract:**

**Purpose:**

Amyotrophic lateral sclerosis (ALS) is a rapidly progressive neurodegenerative motor neuron disease (MND) with heterogeneity in disease onset, progression and treatment response. The Strategic ALS Australia–Systems Genomics Consortium (SALSA-SGC) was established in recognition of the need for large data sets of clinical data matched with biological samples to enable and foster ALS research and better understanding of aetiology and biological mechanisms. SALSA-SGC brought together the major Australian MND clinics to set up sustainable infrastructure that could facilitate long-term human ALS research and clinical trials nationally and internationally.

**Participants:**

Between April 2016 and December 2024, SALSA-SGC recruited 1813 participants, including 1386 ALS/MND cases, 388 controls and 39 others (asymptomatic relatives and ALS mimics). Clinical data and biospecimens are available for 1333 and 1189 ALS cases, respectively, with longitudinal data spanning 4442 total clinic visits and 3201 samples. An open-access online data explorer showcases collected datasets.

**Findings to date:**

Detailed clinical and questionnaire data allow an in-depth description of the cohort, informing clinical and health policy research. Screening for known ALS large-effect risk variants identified 125 mutation carriers (11.5% from N=1059), including 70 with C9orf72 expansions. Single Nucleotide Polymorphism (SNP)-array data (N=1088 cases; N=244 controls) have supported multiple published studies. SALSA-SGC resources are actively used by national and international researchers.

**Future plans:**

Ongoing efforts aim to expand recruitment into regional Australia and enhance sample processing for cell-based studies. The SALSA-SGC resource is accessible by researchers under agreements governed by participant consent, human ethics committee guidelines and agreed use of data and samples.

**STRENGTHS AND LIMITATIONS OF THIS STUDY**

- SALSA-SGC has implemented a flexible governance model which supports both site-specific and cross-consortium collaborative projects, including clinical trials and third-party partners.
- SALSA-SGC has longitudinal clinical data and biological samples that track the variability in disease progression.
- The SALSA-SGC framework is scalable, enabling inclusion of additional MND clinics from smaller centres as funding allows.
- The SALSA-SGC framework has been designed to help support clinical trials.
- The main limitation is that SALSA-SGC only recruits from participating ALS centres across Australia and is not a national registry of people with ALS.

## Introduction

 Amyotrophic lateral sclerosis (ALS) is the most common and malignant form of motor neuron disease (MND), has a complex genetic architecture and is heterogenous in site of onset, spread, progression and response to available therapies. The disease results in a rapidly progressive neurodegeneration of both lower and upper motor neurons with death usually within 3–5 years of disease onset. Given this, short survival time contributes to a point prevalence that makes ALS seem relatively rare compared with other complex diseases, ~8.7 in 100 000 Australian population,^1^ which is slightly higher than the median (IQR) prevalence (/100 000 population) of 5.40 (4.06–7.89) in Europe.^2^ However, based on the lifetime risk of disease ~0.3%,^3^ ALS should not be considered a rare disease and there remains an urgent need to design more effective treatments.

About 10% of people diagnosed with ALS are from families with multiple affected individuals.^4^ Through these families, DNA variants associated with very high risk of ALS have been identified and the genes whose function is impacted by these variants, such as *SOD1, TARDP, FUS, C9orf72,* are a key focus of ALS research.^5^ However, the vast majority of people diagnosed with ALS do not come from such families and are traditionally referred to as ‘sporadic’ cases. Despite not having a family history of ALS, genetic factors will still play a role, as they do with almost every disease. In 2015, Motor Neuron Disease Research Australia (MNDRA) offered a one-off AU$1M grant to support research in sporadic ALS afforded through community support of the 2014 viral ‘Ice-bucket challenge’ fundraiser. This grant enabled the establishment of the Strategic (formerly Sporadic) ALS Australia Systems Genomics Consortium (SALSA-SGC) with a goal to recruit all people living with ALS or related MNDs in Australia into research. Subsequently, SALSA-SGC has received financial support from multiple sources (see funding section). Building on the strong and collaborative research base that already existed in Australia, SALSA-SGC brought together seven major MND clinics to enable consistent clinical and biological collections, under the philosophy that large data sets with a depth and breadth of data fields are needed to answer critical questions related to the cause, progression, symptom onset/spread and treatment tolerance.

The long-term vision of SALSA-SGC was to set up foundational infrastructure integrating clinical, lifestyle and biological information to underpin ALS research in Australia and facilitate a role in international studies. Since its establishment and initial funding, SALSA-SGC has been able to achieve five main aims: (1) Facilitate harmonised data collection and biological samples across Australia; (2) Support detection of genetic and epigenetic risk factors and disease biomarkers, locally and internationally; (3) Build methods and resources that could be sustained and expanded over time and that can support research locally and internationally; (4) Support clinical trials and (5) Enable participants who contribute to SALSA-SGC to gain directly from their enrolment. The name ‘systems genomics consortium’ placed a flag for the long-term vision that clinical data would be matched with multiple layers (ie, across systems) of biological data (genome, epigenome, transcriptome, proteome, lipidome, metabolome) collected from multiple human biosamples (such as blood, urine, faeces, CSF, biopsies). In the initial phase, the focus has been on the collection of longitudinal blood samples matched with clinical data. SALSA infrastructure has been used for independent substudies where data and/or samples are shared back with SALSA-SGC.

Several international ALS resources complementary to SALSA-SGC exist, each with distinct goals. Project MinE^6^ (based in the Netherlands) aims to collect whole genome sequence (WGS) data from 15K cases and 7.5K controls to better understand both the common and rare genetic variation contribution to ALS.^7^ AnswerALS (based in the USA) has clinical, biometric and ‘omic data on induced pluripotent stem cell (iPSC)-derived motor neurons for 1K patients.^8^ Target ALS (USA/international) provides a collaborative framework for sharing high-quality post-mortem tissues, biospecimens and multiomics datasets to support translational research. The European PRECISION ALS project collates patient data across multiple sites (20K cases in 2023) but does not hold linked biological samples.^9^ SALSA-SGC is suitably placed to contribute to these global resources, combining rich longitudinal clinical data together with biobanked biological samples to enable the generation of new data to advance ALS research in the decades ahead.

Here, we describe the SALSA-SGC cohort and follow the Strengthening the Reporting of Observational Studies in Epidemiology reporting guidelines^10^ for cohort studies (online Supplement table 1).

## Cohort description

SALSA-SGC is a prospective, multicentre study collecting longitudinal data and samples across Australian individuals living with ALS, asymptomatic known-mutation carriers and healthy controls. Recruitment started in April 2016 and is ongoing. This cohort profile report has a data censor date of 31 December 2024.

### Sites and governance

**Figure 1:**
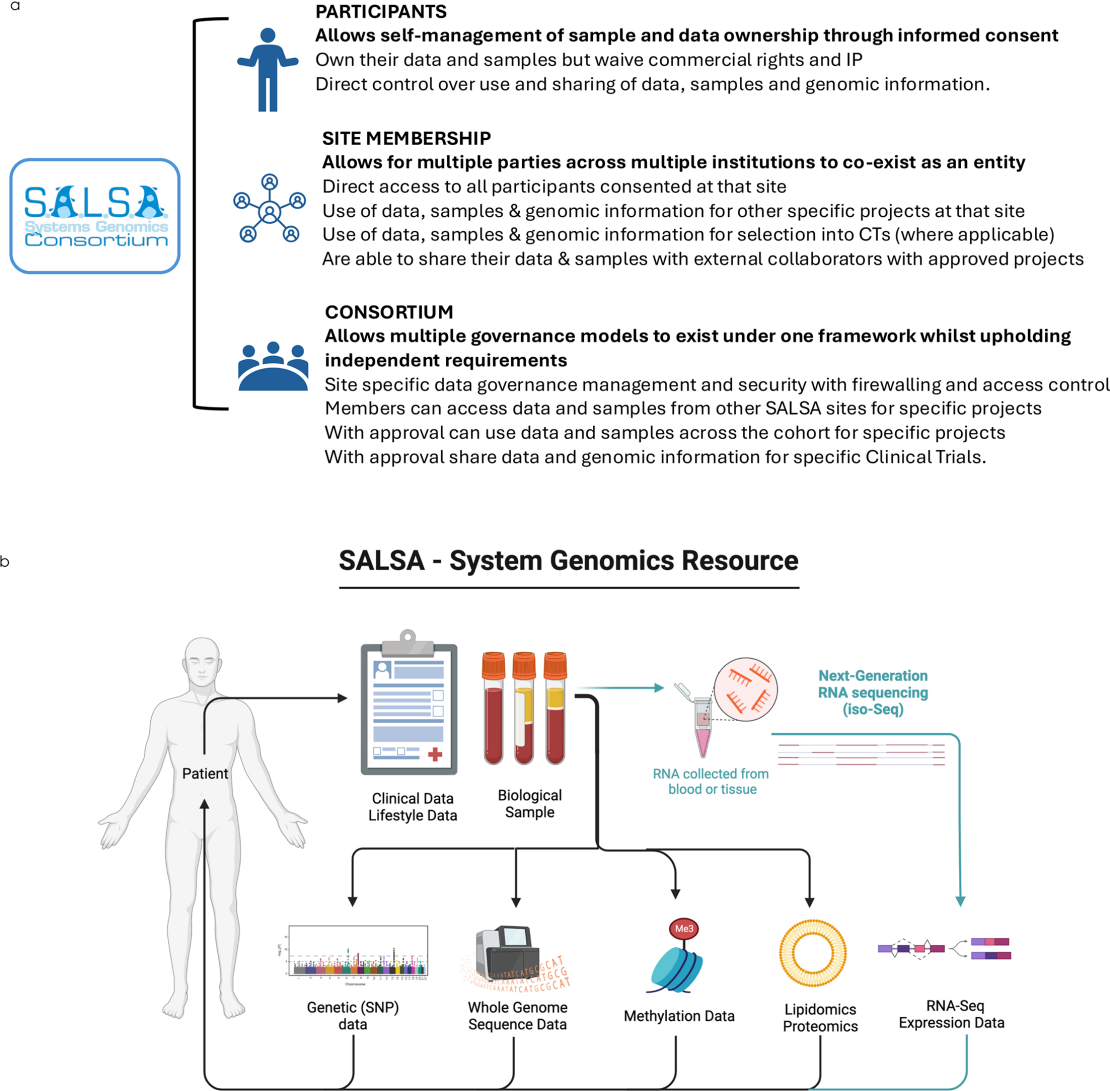
SALSA consortium (A) Research Governance Framework (B) Data Collection schematic. CT, clinical trial; IP, intellectual property; SNP, single nucleotide polymorphism; SALSA, Strategic Amyotrophic Lateral Sclerosis Australia.

SALSA-SGC recruits from major MND clinics across five Australian states: Royal Brisbane and Women’s Hospital (RBWH), Queensland (QLD); Calvary Health Care Bethlehem (CHCB), Victoria (VIC); Flinders Medical Centre (FMC), South Australia (SA); The Perron Institute for Neurological and Translational Science (PI), Fiona Stanley Hospital (FSH), Western Australia (WA); Macquarie University hospital neurology clinic (MQC), Brain and Mind Centre (BMC), Neuroscience Research Australia (NeuRA) (NA), Brain and Nerve Research Centre, Concord General and Repatriation Hospital (CH), New South Wales (NSW). SALSA-SGC launched at each site following local human ethics approvals and with site-specific protocols adapted to the local operational environment. For example, MQC sees patients referred by general practitioners providing care from diagnosis to death. In contrast, CHCB is a tertiary clinic receiving on-referrals from other hospitals and offers multiple medical specialities relevant to ALS care in a single centre. In Australia, MND neurologists work across the private and public healthcare systems, SALSA-SGC clinics operate in public hospitals, but private patients can be linked into ALS research clinics. A robust governance framework and consortium model (figure 1A) was established with each site retaining ownership of primary data, biological samples and derived data but logistically managed centrally by the Human Studies Unit (HSU), Institute for Molecular Bioscience, University of Queensland (UQ), Brisbane.

### Data infrastructure

Participant recruitment, clinical data capture and biological sample tracking is supported centrally through a research portal custom built and maintained by the HSU using the open-source Drupal content management system. Drupal modules and management architecture were implemented specifically to facilitate the SALSA-SGC governance structure and collection protocols with full consideration of Australian Privacy Principles (April 2014), and where possible meeting international data management guidelines (ie, the European General Data Protection Regulation policy). Features include real-time data capture, bespoke data views and downloads, and upload of data collected through other data capture portals. Data access is restricted through multifactor authentication and site/role/user-based access privileges are managed to comply with research governance requirements specific to this consortium. Data are held on UQ computing servers with strict state-of-the-art security measures in place (firewall, PROXY configurations, SSL certificates, SELinux security modules, etc). Data collected through other data portals, for example, MiNDAUS,^4^ are automatically uploaded in bulk to the SALSA-SGC research portal.

### Laboratory infrastructure

Biological collections are managed across three SALSA dedicated laboratories (HSU, MQC and PI). All sites used standardised and audited protocols for the processing of whole blood samples and the fractionation to specific blood derivatives (plasma, buffy coat and serum) for long-term storage. Biological samples collected from CHCB, FMC, RBWH, BMC, NA and CH are shipped to HSU for processing. At HSU, a laboratory and information management system stores information attributed to each biological sample and its derivative, each tracked with data matrix technology bar-codes. HSU has held international accreditation for sample biobanking since 2023.

### Patient and public involvement and engagement

There is strong support for SALSA-SGC from the patient community who recognise the value of ‘big data’ and of their legacy contribution. Given close contact with patients at recruiting sites, feedback from patients has been incorporated into all aspects of data collection. Questions specifically around access and support for care management plans were provided by MND Australia, allowing us to make comparisons across states. Ongoing patient involvement is managed through local community support groups such as those operated by charities like Motor Neurone Disease Australia, its affiliated state MND associations and the MND and Me Foundation.

### Participant eligibility

SALSA-SG recruits cases, healthy controls and asymptomatic carriers of known large effect variants (‘mutations’). All participants must be over the age of 18 and able to provide informed consent. Cases are recruited if a consultant neurologist has provided a suspected or confirmed diagnosis of ALS (or other MND) using clinical guidelines such as the revised El-Escorial criteria.^11 12^ Given the recognition of the blurred boundaries between familial and sporadic ALS,^13^ all cases are recruited. Diagnosis is not always straightforward and some people attending neurology clinics recruited into SALSA-SGC may not end with a final ALS diagnosis. However, these patients are also valuable for studies of biomarkers of disease to discriminate cases from potential ALS mimics. Non-MND ‘healthy’ controls are recruited at some sites and can include non-biological relatives and carers. Currently, asymptomatic carriers of known ALS mutations are recruited at some sites (under additional approved human ethics protocols).

### Recruitment method

All participants to date have been recruited through the multidisciplinary MND clinics (that serve as SALSA-SGC sites) by the treating neurologist after confirmation of eligibility to participate. These clinics are a mixture of primary and tertiary referral centres. Patients presenting at a primary neurology clinic usually require a diagnostic work-up and are, on average, approached within 3 months of a formal diagnosis. Participants presenting at tertiary clinics have been referred there after diagnosis and so are approached immediately for recruitment. The consulting neurologist decides if a patient is physically and psychologically able to participate. The participant information and consent form (online supplemental material 1) covers clinical data capture and biological sample collection every 3 months. The consent includes that biological samples, DNA, clinical and self-reported data can be made available to researchers in future, including commercial entities. Participants are approached only after their initial diagnosis to reduce burden on individuals after receiving a terminal diagnosis. Participants can withdraw from the study at any time but with retention of data already collected/generated. The overview of the SALSA-SGC protocol is summarised in figure 1b.

### Clinical data

Clinical data and biological samples are collected during standard clinic (or at some sites research clinic) visits approximately every 3 months for as long as participants are able. For healthy controls, a single baseline sample is collected alongside some general demographic, biometric and family history information. For asymptomatic mutation carriers, blood collection protocols vary by site, with baseline samples contributed to SALSA. All participant collections are coordinated by clinical research nurses at each site working in with the natural regime of each clinic environment. Data collected at clinical visits and relating to biological samples are entered by the clinical research nurse into the centrally managed data portal. All data collection fields were jointly developed and agreed on by the consortium neurologists and harmonised with ENCALS^14^ protocols. The clinical variables allow derivation of El-Escorial criteria,^12^ Gold Coast criteria,^15^ King’s staging^16^ and ALS Functional Rating Scale-Revised (ALSFRS-R)^17^ subscores. The approved study protocol did not routinely include collection of data associated with respiratory function, cognitive assessments and other clinical information generated as part of clinical care. However, all participants were consented for data sharing of clinical data generated as part of their care and disease management plans. These were uploaded as reports to the centralised MND research portal so they could be accessed and/or used in downstream analyses. Not all participating centres had access to undertake these additional clinical tests and so were not included in the core protocol of the SALSA-SGC.

### Self-report questionnaire data

Some sites administer a lifestyle and environmental exposures questionnaire (online supplemental material 2), which collects information on residential history, education and occupation, family, comorbidities and medications, substance use, physical activity and diet, exposure to hazards, women’s health, and an option for participants to include any extra information they feel relevant to their diagnosis. This questionnaire was designed by the HSU team and implemented using the LimeSurvey platform. It is administered once and takes 30–40 min to complete. Wherever possible, questions were built on existing questionnaires or were designed for benchmarking with other data, for example, the Australian National Census, SOPHIA.^18^

### Biological collections

Biological samples are collected on-site by clinical research nurses or trained phlebotomists and couriered to the clinic-associated laboratory for receipt and processing. The standard SALSA-SGC protocol is a 30 mL blood sample, but participants may also provide saliva where a blood sample is not possible. Blood derivatives such as plasma, buffy coat and serum are barcoded for downstream projects and applications. Blood samples (or extracted DNA) from PI, FSH and MQC are periodically shipped to the central SALSA-SGC lab at HSU for genetic analyses and other researcher-led studies. Other biological samples (urine, stool, RNA) are collected and stored for a subset of samples funded through connected, collaborative studies.

### Genetic and omic data

Extracted DNA is routinely genotyped at the HSU laboratory on the Illumina GSAMD v3 SNP array with an additional 11K SNPS selected by HSU. These additional SNPs include SNPs located within genes known to be involved in neurodegenerative disorders, including 194 variants from ALS-associated genes reported as ‘*pathogenic*’ or ‘*likely-pathogenic’* in the ClinVar database.^19^ The SALSA-SGC routine protocol also includes screening for the pathogenic hexanucleotide expansion in *C9orf72*. This expansion is typically found on a haplotype tagged by the A allele of SNP rs3849942.^20^ Participants who carry the A allele at this locus are screened for the six base-pair expansion using the repeat primed PCR method.^21^

Genotypes of known ALS variants/mutations are returned to the neurologist at each site in both individual patient PDF report and clinic cohort bulk download formats via the SALSA research portal. The genome-wide genotype data are processed through the HSU in-house QC and imputation pipeline to facilitate downstream research (eg, genetic association analysis and polygenic risk scores). SALSA-SGC also provides plasma, serum, etc to a range of genomic projects. Data from these linked genomic projects are included back into SALSA-SGC expanding the depth of the resource available to researchers.

## Findings to date

### Cohort characteristics

**Figure 2:**
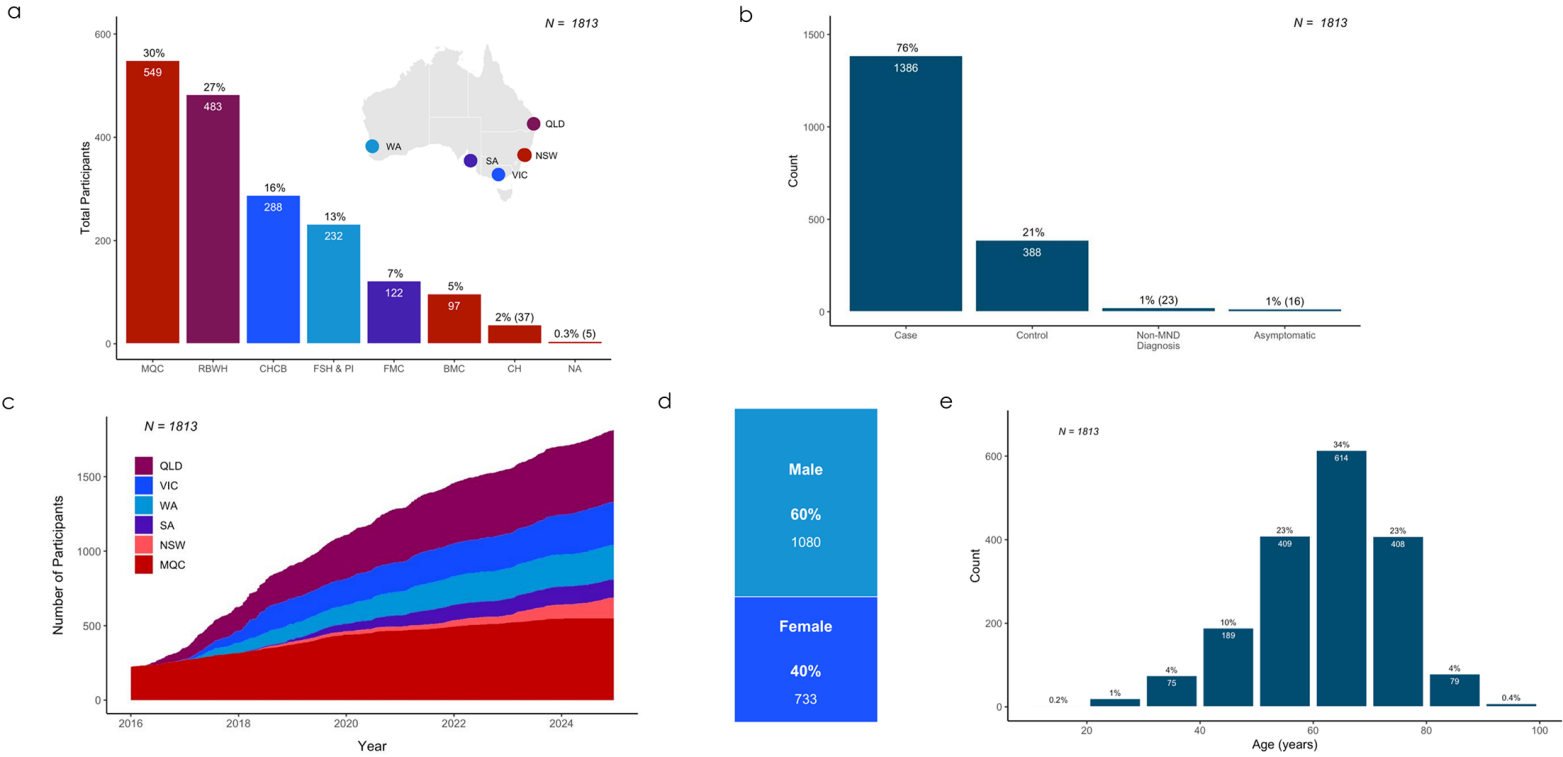
Cohort characteristics. In total, 1813 participants have been recruited into SALSA-SGC described by (A) recruitment sites Royal Brisbane and Women’s Hospital (RBWH), Queensland (QLD); Calvary healthcare Bethlehem, Victoria (CHCB, VIC); Flinders Medical Centre (FMC), South Australia (SA); The Perron Institute for Neurological and Translational Science (PI), Fiona Stanley Hospital (FSH), Western Australia (WA); Macquarie University (MQC), Brain and Mind Centre (BMC), Neuroscience Research Australia (NeuRA) (NA), Brain and Nerve Research Centre, Concord General and Repatriation Hospital (CH), New South Wales (NSW). (B) Participant type. (C) Year of recruitment. (D) Biological sex. (E) Age. SALSA-SGC, Strategic Amyotrophic Lateral Sclerosis Australia–Systems Genomics Consortium.

In total, 1813 participants were recruited into SALSA-SGC from 06 April 2016 to 31 December 2024. This cohort can be summarised by recruitment clinic (figure 2a), by participant type (figure 2b), over time (figure 2c), by biological sex (figure 2d) and age at recruitment (figure 2e). The cohort includes 1386 MND cases (64% male), of which 1333 have clinical data (online supplemental table 2) comprising a total of 4442 clinical visits (each at least 2 months apart). Given that data are collected in real-clinic settings, complete data cannot always be achieved; the numbers presented in the Figures quantify this. Clinics vary in their ability to follow the complete SALSA-SGC protocol; research clinics are more able to allocate time needed for data collection, particularly longitudinal data. Of those invited to participate, it is estimated that the vast majority (90%) provide consent. The most common reason to decline is for patients who live in regional and remote parts of the country and only attend a specialised ALS clinic for the purposes of a diagnosis. In these instances, follow-up care and management is undertaken via telehealth and local health practitioners not involved in the SALSA-SGC. Hence, SALSA-SGC cannot be considered a complete registry of ALS in Australia.

**Figure 3:**
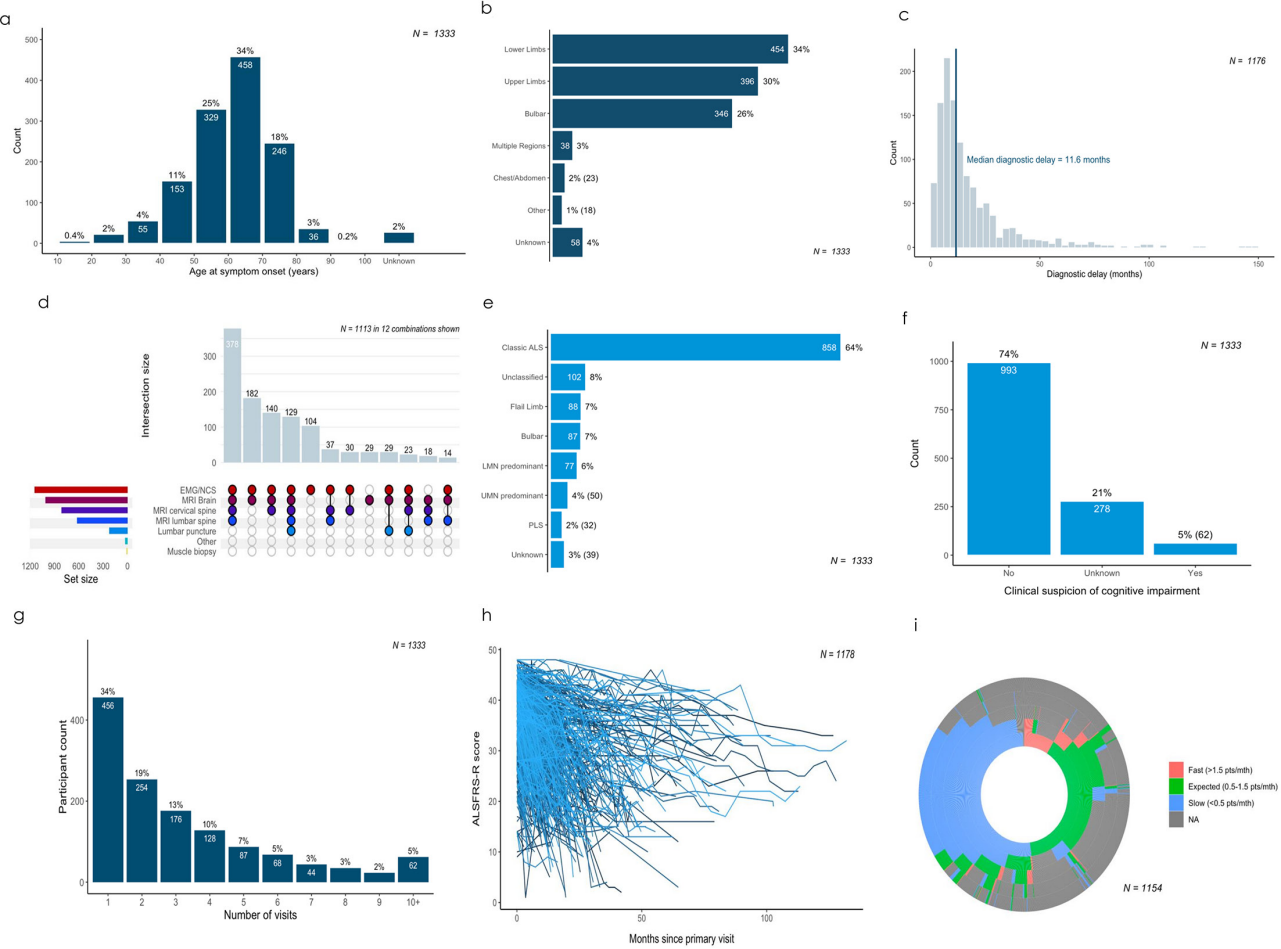
Case cohort characteristics. Described by (A) age of onset of symptoms; (B) region of the body with first symptom onset; (C) diagnostic delay in months from symptom onset to diagnosis; (D) upset plot of tests conducted to assist diagnosis; (E) clinical phenotype at the primary clinic visit; (F) clinical suspicion of cognitive impairment; (G) longitudinal data described through histogram of number of clinical visits (minimum 2 months between visits); (H) ALS Functional Rating Scale-Revised (ALSFRS-R) relative to months since primary visit; (I) sunburst visualisation of disease progression. Each thin slice represents an individual and each ring is a year from the primary clinic visit with 5 years of data represented. The first (inner) ring represents the first year of data collection. The different colours indicate the disease progression rate that the participant is experiencing in each year derived from changes in the ALS-FRS score. The grey represents censorship (ie, non-attendance, presumed death). ALS, amyotrophic lateral sclerosis; EMG/NCS, Electromyography and Nerve Conduction Studies; LMN/UMN, lower/upper motor neuron; NA, not available; PLS, primary lateral sclerosis.

Primary clinic visit reports include age of symptom onset (mean 60.6 years SD 12.2 years, IQR 53.6–69.1 years figure 3a), region of the body with first symptom onset (figure 3b), diagnostic delay (median 11.6 months, IQR 7.0–21.0 months, from symptom onset to diagnosis (figure 3c), the suite of tests completed to assist diagnosis (figure 3d), clinical phenotype reported at the baseline clinical visit (figure 3e) with 67% meeting Gold Coast criteria for ALS, and clinical suspicion of cognitive impairment 5% (figure 3f). Cohort characteristics are described in online supplemental table 3. The lifestyle questionnaire is only administered at some clinics, and to date has been completed by 214 participants (online supplemental table 4).

**Figure 4:**
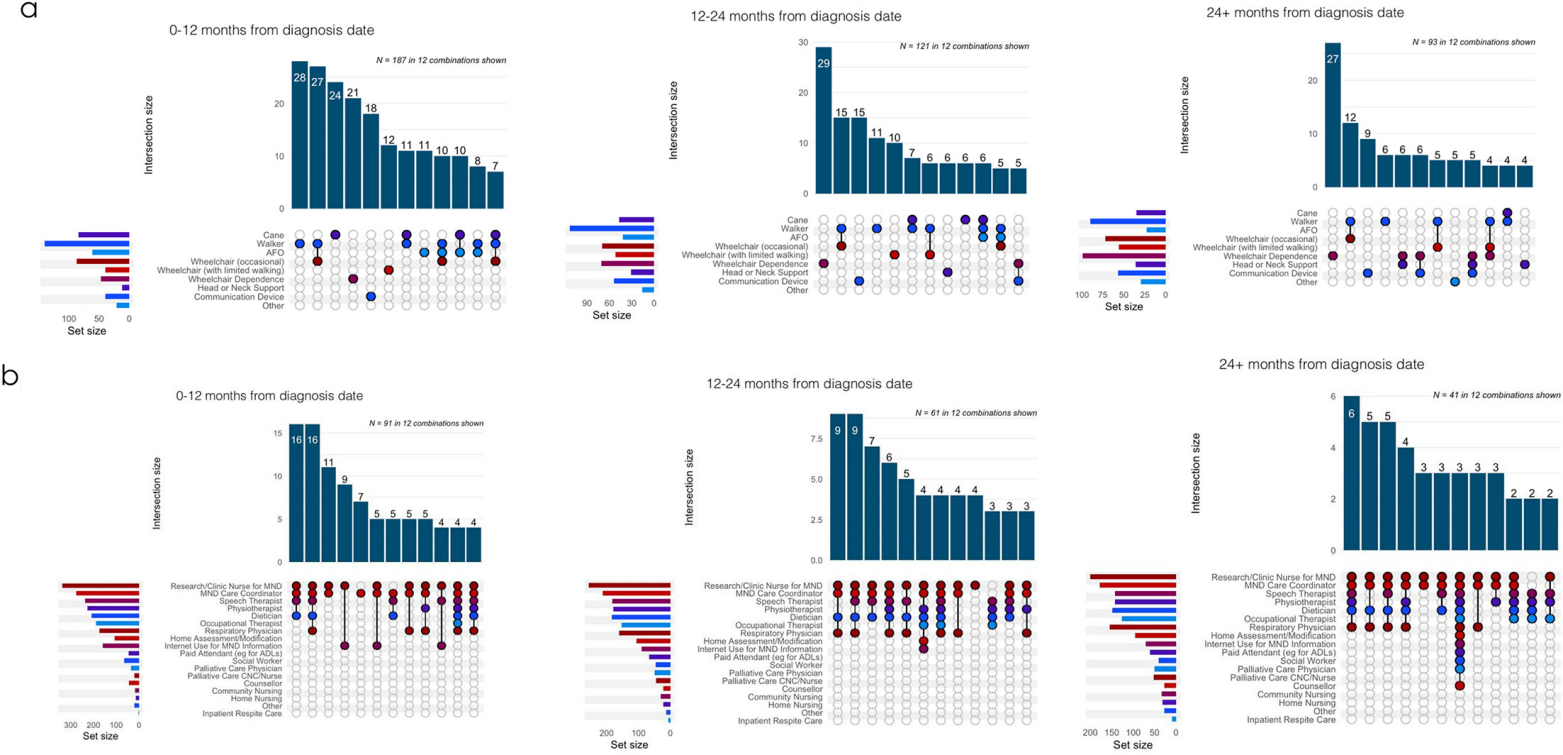
Case cohort healthcare utilisation. (A) Upset plots of use of adaptive devices. (B) Upset plots of health resource utilisation with the three plots representing 0–12 months since diagnosis (left panels), 12–24 months since diagnosis (middle panels), 24+ months (right panels). ADL, Activities of Daily Living; AFO, Age at first onset; Age at first onset; CNC, Clinical Nurse Consultant; MND, motor neuron disease.

Through longitudinal clinic visits, the disease progression can be tracked. The number of clinic visits (median 2, IQR 1–4, figure 3g) is correlated with survival (defined as months since diagnosis). Disability can be tracked across gross motor tasks, fine motor tasks, bulbar function and respiratory function through the ALS-FRS-R (figure 3h). The rate of change of ALS-FRS-R can be classified as slow or fast relative to average progression^22^ (figure 3i). A subset of SALSA-SGC participants has completed questionnaires that allow longitudinal tracking of disability through devices (figure 4a) and healthcare utilisation (figure 4b).

### Biological samples and derived data

Of 1813 participants and 1386 cases recruited into SALSA-SGC, 1497 and 1189, respectively, have provided biological samples (1160 with both clinical data and biological samples).

**Figure 5:**
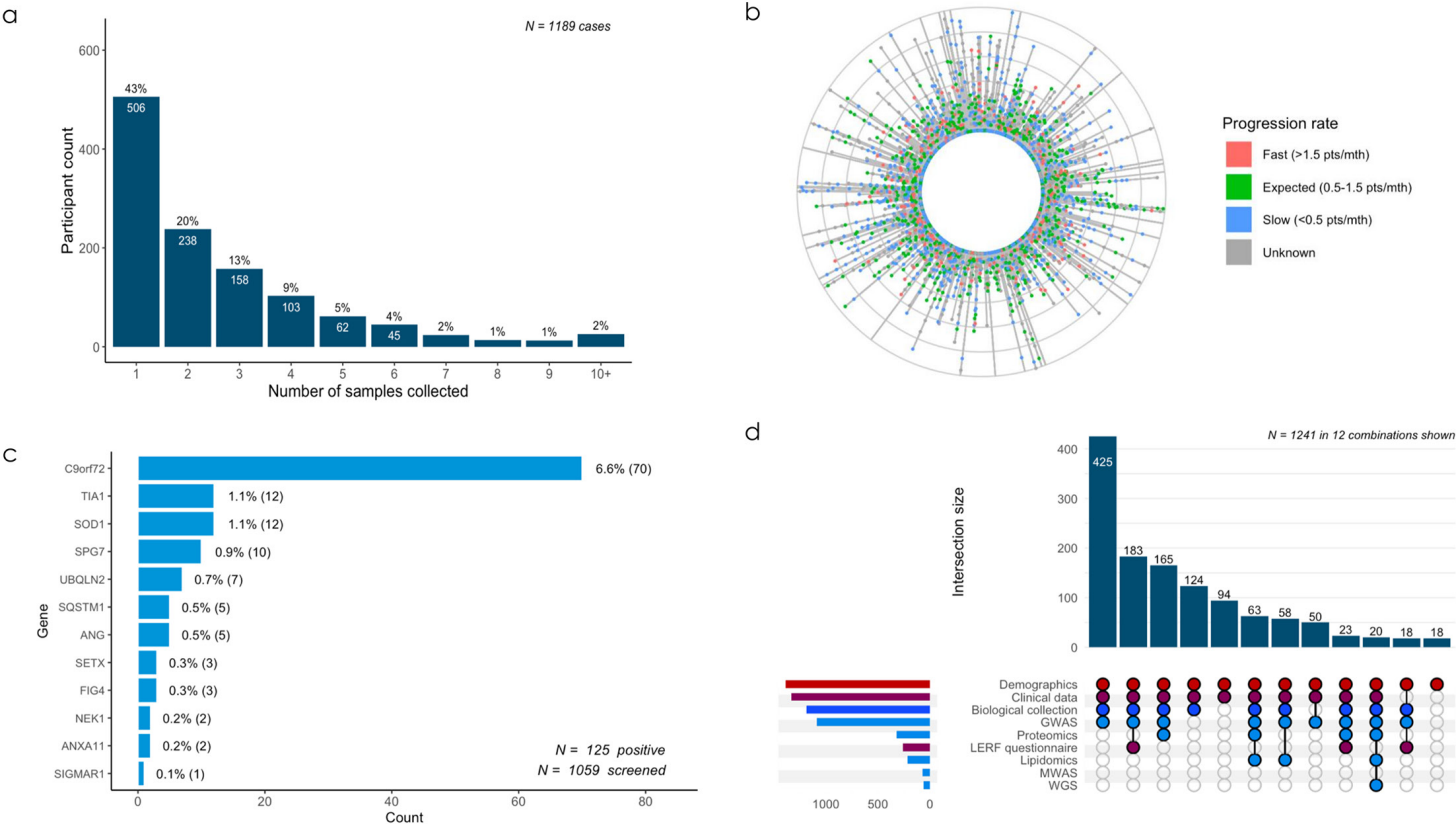
SALSA-SGC biological samples and mutation screening. (A) Histogram of the number of blood samples provided by individuals (>2 months apart). (B) Sunburst plot of blood sample collections. Each grey line represents one participant, and the dots on the line indicate their sample collections. The distance of the dots from the centre ring (first blood sample collection) shows the timing of the sample collection, with each outer ring indicating 1 year from the initial collection. The colour of the dots shows the rate of ALSFRS-R progression at each sample time point (expected rate: 0.5–1.5 ALSFRS-R pts/month). This plot only includes the first 5 years of collection, although some participants do have samples collected over longer periods (C) mutation screen of variants reported as pathogenic or likely pathogenic in the ClinVar database (n=1059 tested). (D) Upset plot of omics data (bar graph truncated to combinations with >15 participants). ALSFRS-R, Amyotrophic Lateral Sclerosis Functional Rating Scale–Revised; GWAS, genome-wide association study; MWAS, Methylome-wide association study; SALSA-SGC, Strategic ALS Australia-Systems Genomics Consortium.

Linked to the 4442 total clinic visits are 3201 biological sample collections. The median time from diagnosis to first biological sample is 6.4 months (IQR 3–14 months), the median sampling delay from disease onset to first biological sample is 20.0 months (IQR 13.0–36.8 months), and 57% provide more than one sample (figure 5a). Within an individual, biological samples can be matched to clinical phenotype and stage of disease and annotated to slow, expected or fast disease progression over time using the ALSFRS-R (figure 5b).

Of 1059 cases screened for known MND mutations, 128 mutations were detected (11.5%, in 125 people). The majority of these were identified as carriers of the repeat expansion in the *C9orf72* gene (greater than 25 repeats of the 6 base-pair signature) (70 people, (6.6%)) (figure 5c). The SALSA-SGC resource includes SNP-array (genome-wide association study, GWAS) data (N_case_=1088 N_control_=239) and a small number of samples with WGS (Ncase=105) and/or DNA methylation data (N_case_=175, N_control_=73). Independent add-on studies have generated cell-free DNA (N_case_=152, N_control_=152),^23 24^ lipidomics data (N_case_=217, N_control_=38) and OLINK proteomics data (N_case_=319, N_control_=75) (figure 5d).

### Published studies

SALSA-SGC genotype data were included in the international collaboration GWAS of ALS ^7^(in which 15 common risk loci were identified) and were also used to investigate polygenic risk score associations.^25^ WGS data have been contributed to Project MiNE^6^ (together with QLD samples collected prior to the start of SALSA-SGC and characterised through WES data.^26^ Other studies have used SALSA-SGC data to focus on specific gene loci *ACSL5-ZDHHC6*,^27^ *STMN2*^28^ and *GPX3/TNIP1*.^29^ Genotypes of the *UNC13A* SNP rs12608932 are being used to select patients into a clinical trial, promoting national screening and patient recruitment.^30^ Cell-free DNA levels are found to be higher in ALS cases than controls with DNA methylation markers indicating that the circulating cell-free DNA is derived from muscle cells.^23^,^25^ DNA methylation data have contributed to Methylome-wide association study (MWAS) of ALS^31 32^ implicating metabolic, inflammatory and cholesterol pathway signatures in those with ALS and shared associations across neurological disorders,^33^ although many DNAm samples were recruited prior to SALSA-SGC and are not listed in our summaries. Collaborator projects on lipidomic, transcriptomic and proteomic data analyses and an iPSC resource project are all currently underway.

### Future directions

Future directions for SALSA-SGC include continued data collection under a flexible governance model (online supplemental material 3), contingent on grant funding, with current applications seeking to: (1) support ‘postal’ enrolment/sample collection to remove barriers for those with MND living in remote and rural Australia and (2) support blood processing protocols for storage of peripheral blood mononuclear cells (PBMCs) facilitating future generation of lymphoblastoid cell lines, iPSC and organoid models. In parallel, existing data and samples are being actively shared and integrated with international consortia to accelerate large-scale discovery efforts. This includes generation of RNA-seq data in blood (N_cases_=121, N_controls_=60) and novel blood biomarker investigations (ie, profiling of extracellular vesicles in blood, Somalogic proteomics) paired with matched benchmarking against established marker/methods such as plasma neurofilament light chain using single molecule arrays (Simoa-Quanterix). The flexible governance structure allows for multiple parties across multiple institutions to co-exist as an entity while undertaking site-specific research objectives.

### Strengths and limitations of this study

A key strength of the SALSA-SGC approach is its flexible governance model (online supplemental material 3) which supports both site specific and cross-consortium collaborative projects, including clinical trials and third-party partners. The framework is also scalable, enabling inclusion of additional MND clinics from smaller centres as funding allows. The framework is designed to support clinical trials. The main limitation is that SALSA-SGC only recruits from participating ALS centres across Australia and is not a national registry of people with ALS. Some clinical sites are more able to support data collection for research than others. Missing data are mostly site dependent and are not a reflection of patient characteristics. Patients living regionally are under-represented (because their attendance at major ALS clinics in metropolitan sites is limited). To our knowledge, missing data is unlikely to be biased by other patient characteristics, except that patients presenting at an advanced disease stage are less likely to be recommended to participate in SALSA-SGC by their consulting neurologist. We hope to expand recruitment to include all individuals living with ALS in both metropolitan and regional areas, which would facilitate improved evaluation of health services by geography. Another key strength of SALSA-SGC is the longitudinal clinical data and biological samples that track the variability in disease progression. The underlying vision was to build a systems genomics resource to benefit those affected by ALS, and while this has been partially realised, ongoing efforts aim to integrate multiple layers of omics data to support research translation.

### Collaboration and data availability

Guided by the participant consent process, SALSA-SGC actively encourages the use of this resource by the research community to accelerate research towards prevention and treatments of ALS. The consortium operates under an approved governance framework, including authorship guidelines (online supplemental material 3) and encourages that data generated using the resource be made available to other researchers, subject to necessary de-identification and ethical requirements. Data can be explored through a purpose-built online data explorer (https://salsasgc.org/explore), which generates dynamic versions of the Figures presented in this paper. The explorer tool enables queries by key filters such as age, gender, gene mutation status and clinical variables. Queries can be saved and submitted alongside formal data access application. All requests are reviewed by a scientific committee, and before the data are made available, appropriate research governance agreements are established. Access to data and/or samples is available to bona fide research and commercial organisations, with biological samples use subject to approval by the relevant site neurologist/sample owner. Some published GWAS and MWAS data have been deposited in dbGAP (phs002068.v1.p1). Access to standard operating procedures, laboratory protocols and analysis scripts is available on request.

## Supplementary material

10.1136/bmjopen-2025-110906 online supplemental material 1

10.1136/bmjopen-2025-110906 online supplemental material 2

10.1136/bmjopen-2025-110906 online supplemental material 3

10.1136/bmjopen-2025-110906 Online Supplement Table 1

10.1136/bmjopen-2025-110906 Online Supplement Table 2

10.1136/bmjopen-2025-110906 Online Supplement Table 3

10.1136/bmjopen-2025-110906 Online Supplement Table 4

## Data Availability

Data are available on reasonable request.
