# Supplementary material for: Strategic Amyotrophic Lateral Sclerosis Australia–Systems Genomics Consortium (SALSA-SGC): cohort profile
Source: BMJ Open. 2026 Jun 18;16(6):e110906. doi: 10.1136/bmjopen-2025-110906 (PMC13288974; doi:10.1136/bmjopen-2025-110906)
Supplement: online supplemental material 1 [file bmjopen-16-6-s001.pdf]

Participant Information Sheet/Consent Form

Genetic Study – Adult providing own consent

Title: *System Genomics in Amyotrophic Lateral Sclerosis (ALS) – SALSA_SGC Project*
Short Title: *SALSA Project*
Local Principal Investigator: *Professor Steve Vucic*
Lead Investigators: *Prof Naomi Wray*
A/Prof Ian Blair
Location: *Concord Repatriation General Hospital*

1 Introduction

You are invited to participate in a research project conducted by Professor Steve Vucic and colleagues from Concord Hospital as part of the Sporadic ALS Australian Systems Genomics Consortium (SALSA: www.salsasgc.org). The SALSA consortium comprises of ALS clinicians and researchers from around Australia and Europe who will collect clinical data and biological samples using an agreed set of protocols to better understand the causes of MND. This study is being funded by Fight MND and regular reports about this study's progress is shared with them . We are inviting people with a diagnosis of Amyotrophic lateral sclerosis (ALS, the most common form of motor neuron diseases) both sporadic and familial, to participate.

The purpose of this research is: To identify genes that may be linked to ALS and to investigate the mechanisms of ALS using molecular biomarkers (biological substances that can be detected in the blood which indicate a disease). This is a genetic study and aims to recruit individuals who have been diagnosed with ALS.

This Participant Information Sheet/Consent Form tells you about the research project. It explains what your participation may involve, the benefits and risks associated with your participation and how we will protect the privacy of your information and any biological samples you provide.

Please read this information carefully. Ask questions about anything that you don't understand or want to know more about. Before deciding whether or not to take part, you might want to talk about it with a relative, friend or your local doctor.

Participation in this research is voluntary. If you don't wish to take part, you don't have to. You will receive the best possible care whether or not you take part.

If you decide you want to take part in the research project, you will be asked to sign the consent section. By signing it you are telling us that you:

- Understand what you have read

- Consent to take part in this research project
- Consent to the tests and research that are described
- Consent to the use of your personal and health information as described

You will be given a copy of this Participant Information and Consent Form to keep.

2 Who is running this Study?
System Genomics in Amyotrophic Lateral Sclerosis (ALS) – SALSA_SGC Project

Local Principal Investigator:	Professor Steve Vucic<br>Brain & Nerve Research Centre<br>Concord Repatriation General Hospital

Sporadic ALS Australia – Systems Genomics Consortium (SALSA_SGC)

Prof. Naomi Wray:	Lead Principal Investigator<br>Institute for Molecular biosciences<br>University of Queensland, Queensland
A/Prof Ian Blair:	Lead Principal Investigator<br>Macquarie University, Sydney
Ms. Anjali Henders:	Project Manager, University of Queensland

This project is funded by Fight MND (https://fightmnd.org.au/)

3 What is genetic research?
Genes are made of DNA – the chemical structure carrying your genetic information that determines many human characteristics such as the colour of your eyes or hair.

Researchers study genes in order to understand why some people have a certain condition such as Amyotrophic Lateral Sclerosis (ALS) and why some people do not. Understanding a person's genes also may be able to explain why some people respond to a treatment, while others do not, or why some people experience a side effect and others do not.

The aim of genetic research is to improve our understanding of how genes and environmental factors interact to influence the health of individuals and populations. In doing so, genetic research generates knowledge with the potential to improve individual and community health. Specifically genetic research tries to identify genes that may be linked to health or to specific disorders or traits, either physical or mental.

4 What is the purpose of this study?
The purpose of this study is to identify genes that may be linked to ALS and to develop diagnostic biomarkers, which can provide insight into disease onset and its progression. We aim to collect

clinical and self-reported disease information and biological samples from participants with a clinical diagnosis. Specifically we will ask all participants to consent to the provision of blood samples and where this is not possible a saliva sample. Collection of these samples will allow us to use genetic information and develop specialised techniques for specific biomarkers that are involved in biological processes that change over time. This type of genetic and biomarker information can then be used to develop targeted treatment.

We know that ALS is a complex genetic disease and by merging genetic information with clinical information from many affected individuals and comparing it to healthy controls we have the best opportunity to discover new genes and their effects on biological processes involved in the onset and progression of ALS.

Our genetic understanding of ALS is not complete, however information on the causes of ALS that occurs in families has provided information on some of the genetic and biological processes that are involved in this disease. We urgently need to expand this knowledge to improve the detection, prevention and treatment of ALS.

5 What does participation in this research involve?

You will be asked to consent, agreeing to have clinical information collected as part of your routine appointment with your Neurologist, provide blood samples, complete an online questionnaire and grant access to your clinical data in relation to your symptoms, treatment and on-going health care.

<u>Consent</u>

If you agree to participate you will be asked to sign this consent form prior to any data or sample collection.

<u>Clinic Visit</u>

The research team, including your consulting neurologist, will collect information about your ALS diagnosis, general health history, physical measurements (height, weight and girth) and lifestyle such as smoking and your occupation. They will also administer the ALS Functional Rating Scale Revised (ALSFRS-R) interview, which collects information about your physical abilities such as movement, dressing and eating. This will be administered by the research team approximately every three to six months and will coincide with your routine visit to the MND clinic.

<u>Online Questionnaire</u>

Online Lifestyle and Environmental Risk Factors Questionnaire - You will be asked questions about your ancestry, family, occupation, residential history, diet, physical activity, lifestyle behaviours e.g. smoking, alcohol and exposure to chemicals and pesticides.

There are some questions regarding use of prescription and illicit drug as well as medical history information including questions pertaining to sexually transmitted diseases (STDs). Some questions are only relevant to some people, for example questions relating to menstruation and pregnancy.

You will receive a unique research participant number and a link via email to complete the questionnaire online. This will take between 30- 40 minutes to complete and you will only need to complete this questionnaire once at your fisrt SALSA visit. The questionnaire can be completed over several sessons allowing you to pause and complete at a later time.If you are unable to complete questionnaires online, the research team will offer you to complete a paper copy or complete it by telephone interview.

You are not obliged to answer any questions that make you feel uncomfortable or questions you would prefer not to answer. You can skip over them.

Biological Sample

You will be asked to donate a sample of blood (30mL), to collect DNA for genetic analysis. Other parts of your blood sample such as plasma and serum will also be collected and stored. We will ask you to provide a blood sample every three to six months during your routine visit to the MND clinic for as long as you are able. Your blood sample will be collected by qualified research nurses and phlebotomists, and then sent to the Institute of Molecular Biosciences (IMB), University of Queensland, for processing.

Biological samples collected from you will be used to generate genetic information, which will include sequencing your genome (the total of all the chromosomes that contain your genetic information). This information will be screened to look for variations; firstly between people with an ALS diagnosis and healthy controls and secondly compare differences between those with ALS to see if there are any similarities from which we can draw conclusions about disease onset and progression. Asking you to provide a blood sample every three to six months will enable our team to look at changes in biological processes as the disease progresses and see if we can detect and accurately measure them in the blood.

We know that research progresses faster if other scientists can also study your data. Other scientists may obtain access to and use your de-identified health information, genetic information and biological samples for rare disease research. Scientists not involved in the Salsa Consortium who may want to use your data or samples, can only be used by researchers who have their study approved by a Human Research Ethics Committee. Any scientists who wish to use your data must also agree to protect your privacy and store data securely.

This project will generate results that will be presented at conferences and published in scientific journals. This is in accordance with the NHMRC research guidelines under which this study is conducted. Some journals require that we deposit genetic data into secure databases that can be accessed by other researchers who have appropriate approvals. These databases do not store any personal or identifying information only genetic data.

Genetic information from your samples may be shared with other MND researchers both in Australia and overseas if consented, using an Australian based data-transfer platform called CLOUDSTOR. The SALSA-SGC collaborates with the international MND consortium called Project MinE, which analyses clinical and genetic information about ALS. Project MinE is an international, large-scale research initiative devoted to discovering genetic causes of ALS. Only your genetic and clinical information but NOT your personal and identifying information

may be shared with Project Mine and they must store your data under strict European Union data standards called GDPR.

This study will also involve the option to consent for the long-term storage of your blood samples. If you agree to your samples being stored, you will be asked to consent to store your samples in this way.

There are no costs associated with participating in this project and or follow-up medical care required will be covered by this research project.

We do recommend that you discuss your participation in this project with your family and GP before consenting.

6 There are certain risks and discomforts that might be associated with this research
There are risks and discomforts that are part of every study and they deserve careful thought and consideration:

Blood sampling: While giving your blood samples, you may experience some discomfort, bruising and or bleeding at the site of needle insertion. Occasionally, some people experience dizziness or feel faint. In very rare cases some individuals may develop an infection at the venipuncture site.

Discomfort: The online questionnaires will ask personal questions that you may find sensitive, or make you feel uncomfortable depending on your personal experience. The questions are all optional and at any time you may skip a question or stop immediately. You are under no obligation to answer every question.

Breach of Confidentiality: This could occur as a result of many different people being involved in the collection of data and biological samples for this study. Other people could see your data that has been collected, particularly if some parts of your participation have coincided with another research project or clinical appointment.

Someone could get access to your samples or data. This could make it harder for you to get or keep a job or insurance. There are Australian and international laws against the misuse of genetic information, but these laws may not give full protection. We think the risk of this happening is very small, bit it could still occur.

Genetic Information: Someone could trace the coded information in a scientific database back to you and your participation in this study. Even without your name or other identifiers, your genetic information is unique to you (like a fingerprint). We think the risk of this happening is very low.
When conducting genetic studies in families, situations such as non-paternity or previously unknown biological relationships can be discovered. In this study, genetic information will not be released to individuals or shared with other family members. All genetic information will be treated as confidential.

Generating genetic information using the DNA obtained from your sample raises some important issues. Standard practice for genomic research is not to return research findings to participants. However, you can indicate on your consent form whether you would like information relating to the primary aims of this project reported back to your clinician if they are proven to be valid and of health significance to you.

Someone could get access to your samples or data. This could make it harder for you to get or keep a job or insurance. There are Australian and international laws against the misuse of genetic information, but these laws may not give full protection. We think the risk of this happening is very small, bit it could still occur.

Results that could be of significance to you or your family will need to be repeated and the results verified. This will involve you consenting for further genetic testing, having a new blood sample taken and having it tested in an accredited clinical testing laboratory. This is standard practice for research participants receiving information about their genome. Before the results are validated, you will be counselled about the possible issues that might arise and the risks involved for you and potentially your family. This is especially important for individuals who are found to have a genetic mutation that is associated with an increased risk of developing a disease such as cancer or heart disease. If you agree to provide a new sample for genetic testing this will be covered as part of the clinical care being provided at Concord Repatriation General Hospital.

In the event you decide to withdraw from this study your data and samples will be removed from our databases and laboratory and will not be included in any current or prospective work.
Your samples and data that have already been published cannot be recalled. Any data that has been deposited into a public database will be removed through a formal process by providing your unique study identifier to that organization along with the withdrawl request for removal from on-going and/or prospective projects.

7 Compensation for injuries or complications

If you suffer any injuries or complications as a result of this study, you should contact the study doctor as soon as possible, who will assist you in arranging appropriate medical treatment. If you are eligible for Medicare, you can receive any medical treatment required to treat the injury or complication, free of charge, as a public patient in any Australian public hospital.

In addition, you may have a right to take legal action to obtain compensation for any injuries or complications resulting from the study. Compensation may be available if your injury or complication is sufficiently serious and is caused by unsafe drugs or equipment, or by the negligence of one of the parties involved in the study (for example, the researcher, the hospital, or the treating doctor). You do not give up any legal rights to compensation by participating in this study.

8 Will the blood sample be identifiable as mine after it is stored?

No. The stored blood sample will not be identifiable as yours. It will be coded with a unique number which is known only to the researchers working directly on this study.

9 What are the possible benefits from being in this study?

We cannot guarantee that you will receive direct benefit from being in the study, although you may gain some insights into your disease. Society will benefit by the increased understanding that this study will bring in improving diagnosis, treatment or care of people with rare diseases.

All data pertaining to you, including your DNA you have provided (but not your name or address) may be made available to researchers in the future, some of whom may have commercial interests however you will not receive any direct benefit from participating in this type of research. Participants in this or any other related projects cannot claim ownership rights to any medical or scientific product that results from research from their samples.

Research results including genetic information about you as an individual will not be available to you, however in some cases research findings of validated health significance may be shared to your clinician if you have consented to this. Any information we share with them cannot be used in decisions regarding your current or ongoing primary medical care.

We hope that our work will help people with ALS in the future if we increase our knowledge of how genes are involved.

10 How long will your involvement in this study last?

We will ask you to provide a blood sample every three to six months during your routine visit to the MND Clinic for as long as you are able. The questions relating to your day to day activities are normally completed as part of your routine clinical appointment.

Your participation is voluntary and the length of your involvement in this study is at your discretion and under advice from your consulting neurologist. You may choose not to participate in this research study and/or you may withdraw your consent at any time and at any point in the study. To request to withdraw from the study, or that your sample is no longer used for research, you should speak to Professor Steve Vucic, and request that you be withdrawn from the study or that your sample be destroyed. However, any research findings associated with your sample cannot be destroyed or recalled.

Your decision whether to take part or not to take part, or to take part and then withdraw, will not affect your routine treatment, your relationship with those treating you or your relationship with Concord Repatriation General Hospital.

11 What will happen to information and samples collected during the study?

Your genetic code (DNA) will be extracted from the blood sample that you provide. We may extract the whole sequence or just parts of your DNA.

Your clinical and genetic information will be stored on a secure database at the Institute for Molecular Biosciences at the University of Queensland. This database is purpose-built, and the only people who will have access to your individual identity are the Principal Investigators and authorised project staff. Your information will be stored securely and your identity/information

will be kept strictly confidential, except as required by law. Study findings may be published, but you will not be individually identifiable in these publications.

As part of this project we may send genetic information collected from your samples to an international database, which collects and stores information about rare diseases. Only your genetic information but NOT your personal and identifying information will be sent.

Your data and biological samples will be stored indefinitely at the Institute for Molecular Biosciences at the University of Queensland, unless you request to withdraw.

12 How will I know if my samples are being used in the future?

By providing your consent you are allowing us to use your information in future projects. Your data can only be obtained and used by researchers who have their study approved by a Human Research Ethics Committee. We do not know at this stage what these other projects will involve. The HREC will determine whether or not your consent should be obtained at that time for a particular research project. Other scientists may obtain access to and use your de-identified health information, genetic information and biological samples for rare disease research. Any scientists who wish to use your data must also agree to protect your privacy and store data securely.

13 Who will have access to my blood sample once it has been stored?

The custodians charged with ensuring appropriate standards are met in storing and managing the tissue bank will have access to your sample. Researchers involved in research approved by a HREC may also have access to your sample.

14 How will my information be kept confidential?

The Health Service, Principal Investigators and project staff are bound to undertake this research in accordance with the Australian Privacy Principles 2014, the Australian Code of the Responsible Conduct of Research and NHMRC National Guidelines for the Ethical Conduct of Human Research (2007) updated 2018.

They will take all reasonable measures to protect the confidentiality of your records and your identity will not be revealed in any publication that may result from this study. We will keep confidential your name and any other personal information we learn about you. Your personal identifying information will not be given out to anyone. We will take the following steps to ensure confidentiality:

A research code will be assigned to you and your name will not be used. The only people who will have access to your individual identity are the Principal Investigators and authorised project staff. The results from the analysis of your DNA will not be released or shared in any way with your relatives, insurance companies, or any third party not involved in research. Disclosure of your personal information will only occur where it is allowed under Australian Law and with your consent.

15 Complaints and Compensation

All research in Australia involving humans is reviewed by an independent group of people called a Human Research Ethics Committee (HREC). The ethical aspects of this research project have been approved by the HREC of Sydney Local Health District – Concord Repatriation General Hospital.

This project will be carried out according to the National Statement on Ethical Conduct in Human Research (2007) updated 2018. This statement has been developed to protect the interests of people who agree to participate in human research studies.

If you suffer any injuries or complications as a result of this research project, you should contact the study team as soon as possible and you will be assisted with arranging appropriate medical treatment. If you are eligible for Medicare, you can receive any medical treatment required to treat the injury or complication, free of charge, as a public patient in any Australian hospital.

If you suffer any distress or psychological injury as a result of this research project you should contact the study team as soon as possible. They will assist you in arranging appropriate treatment and support.

16 Further information and who to contact

The person you may need to contact will depend on the nature of your query.

If you want any further information concerning this project or if you have any medical problems which may be related to your involvement in the project, you can contact the principal study doctor on 02 9767 8461 or any of the following people:

Clinical contact person

Name	Professor Steve Vucic
Position	Neurologist
Telephone	Mobile: 0415833811
Email	Steve.vucic@sydney.edu.au

If you have any other questions you may also contact the SALSA Project Manager Ms Anjali Henders on 07 3346 6429 or a.henders@uq.edu.au

For matters relating to research at the site at which you are participating, the details of the local site complaints person are:

Complaints contact person

Name	CRGH Research Governance Officer
Position	Research Governance Officer
Telephone	02 9767 6233
Email	SLHD-ConcordEthics@health.nsw.gov.au

If you have any complaints about any aspect of the project, the way it is being conducted or any questions about being a research participant in general, then you may contact:

Reviewing HREC approving this research and HREC Executive Officer details

Reviewing HREC name	SLHD Human Research Ethics Committee
Telephone	02 9767 6233
Email	SLHD-ConcordEthics@health.nsw.gov.au

Local HREC Office contact (Single Site -Research Governance Officer)

Name	CRGH Research Governance Officer
Telephone	02 9767 6233
Email	SLHD-ConcordEthics@health.nsw.gov.au

CONCORD REPATRIATION GENERAL HOSPITAL

Health Sydney Local Health District

Consent Form - *Adult providing own consent*

Title: ***System Genomics in Amyotrophic Lateral Sclerosis (ALS) – SALSA_SGC Project***

Short Title: ***SALSA Project***

Principal Investigator: ***Professor Steve Vucic***

Lead Investigators: ***Prof Naomi Wray***
A/Prof Ian Blair

Location: ***Concord Repatriation General Hospital***

Declaration by Participant

I have read the Participant Information Sheet or someone has read it to me in a language that I understand. I agree to participate and provide information and biological samples as required. I consent to participate in this study under the following conditions:

- I have read all the information about the project and I have had the opportunity to raise questions about my participation and be provided with further information about specific aspects of the study.
- I have agreed to provide biological samples and their use has been explained and accepted by me including the generation of genetic information by sequencing my genome.
- I understand that participation in this project is voluntary and that I am free to withdraw my consent and to discontinue at any time without stating the reason. Refusal to participate or subsequent withdrawal from the project will in no way influence any treatment I receive now or in the future.
- I understand that all data pertaining to me including my DNA I have provided (but not my name or address) may be made available to researchers in the future, some of whom may have commercial interests. I donate my biological sample freely for these purposes and waive any claim to commercial rights arising from this work.
- I give permission for my doctors, other health professionals, hospitals or laboratories outside this hospital to release information to The University of Queensland concerning my condition and treatment for the purposes of this project. I understand that such information will remain confidential
- I understand that, if I decide to withdraw from the study, I may be contacted to discuss genetic information obtained from my sample. Alternatively, a member of the research team may request my permission to obtain access to my medical records for collection of follow-up information for the purposes of research and analysis.

- I understand that if I decide to withdraw from the study, that any research findings that have already been reported or published using data associated with your sample cannot be destroyed or recalled.

- I understand that I will be given a signed copy of this document to keep.

Please select only one option:

☐ YES — I request that genetic research findings of validated health significance identified in my DNA be shared with my clinician and understand that validation of these will require an additional blood sample to be provided. I understand this will be independent from the research study.

OR

☐ NO — I do not want genetic research findings of validated health significance identified in my DNA to be shared with my clinician. I understand this means I will not receive any information through my participation in this project of genetic research findings that may be of importance to me and/or my family.

In respect to the storage and use of my genetic samples, I give permission for the use of my DNA and/or tissue for the purpose of:

1. this research project only	Yes ☐	No ☐
2. this research project and any closely related future research projects (such as Project MinE)	Yes ☐	No ☐
3. future research projects that may or may not be related to this research project	Yes ☐	No ☐

I agree to be recontacted for future HREC approved research projects.	Yes ☐	No ☐

Name of Participant (please print) ______________________________
Signature ______________________ Date ______________________

Under certain circumstances (see Note for Guidance on Good Clinical Practice CPMP/ICH/135/95 at 4.8.9*) a witness* to informed consent is required.*

Name of Witness* to Participant's Signature (please print) ______________________________
Signature ______________________ Date ______________________

* Witness is not to be the investigator, a member of the study team or their delegate. In the event that an interpreter is used, the interpreter may not act as a witness to the consent process. Witness must be 18 years or older.

Declaration by Study Doctor/Senior Researcher†

I have given a verbal explanation of the research project, its procedures and risks and I believe that the participant has understood that explanation.

Name of Study Doctor/ Senior Researcher† (please print) ______________________________
Signature ______________________ Date ______________________

† A senior member of the research team must provide the explanation of, and information concerning, the research project.

Note: All parties signing the consent section must date their own signature.

Form for Withdrawal of Participation - *Adult providing own consent*

**Title:**	***System Genomics in Amyotrophic Lateral Sclerosis (ALS) – SALSA_SGC Project***
**Short Title:**	***SALSA Project***
**Principal Investigator:**	***Professor Steve Vucic***
**Location:**	***Concord Repatriation General Hospital***

<u>Declaration by Participant</u>

I wish to withdraw from participation in the above research project and understand that such withdrawal will not affect my routine treatment, my relationship with those treating me or my relationship with Concord Hospital.

I request that all my biological sample collected and banked be deleted, destroyed or returned to me if it is still identifiable.

Name of Participant (please print) ______________________________

Signature ______________________ Date ______________________

In the event that the participant's decision to withdraw is communicated verbally, the Study Doctor/Senior Researcher will need to provide a description of the circumstances below.

Declaration by Study Doctor/Senior Researcher†

I have given a verbal explanation of the implications of withdrawal from the research project and I believe that the participant has understood that explanation.

Name of Study Doctor/
Senior Researcher† (please print) ______________________________

Signature ______________________ Date ______________________

† A senior member of the research team must provide the explanation of and information concerning withdrawal from the research project.

Note: All parties signing the consent section must date their own signature.
