# Supplementary material for: Strategic Amyotrophic Lateral Sclerosis Australia–Systems Genomics Consortium (SALSA-SGC): cohort profile
Source: BMJ Open. 2026 Jun 18;16(6):e110906. doi: 10.1136/bmjopen-2025-110906 (PMC13288974; doi:10.1136/bmjopen-2025-110906)
Supplement: online supplemental material 2 [file bmjopen-16-6-s002.pdf]

MND Lifestyle and Environmental Risk Factors

Thank you for consenting to take part in our research project. This project aims to understand the effects that the environment, our lifestyle and behaviours have on the onset and progression of ALS. This questionnaire will take you through a series of questions and should take you approximately 35 minutes to complete. All questions are voluntary and you can skip questions if you prefer not to answer them.

Before you start the questionnaire we would like to remind you that you have already provided written consent to participate in this project. If you would like to review the consent form that you signed please click on the following link: Participant Information Sheet and Consent Form.

As a reminder, we will collect and manage your information under the conditions in which you have provided your consent. In accordance with the Australian Privacy Principles 2014, the Australian Code of the Responsible Conduct of Research and NHMRC National Guidelines for the Ethical Conduct of Human Research (2007) guidelines this project will keep confidential your name, contact details and the answers you provide to this questionnaire. Your personal identifying information will not be given out to anyone. Any information you may disclose to us including your answers to sensitive questions (for example, your answers around drug use) will not be disclosed to anyone outside of the research team unless disclosure is required under Australian Law.

If you have decided that you no longer want to take part in this research project, that is perfectly fine. Please click here and you will be exited from this questionnaire and withdrawn from this project. Withdrawal will only affect your participation in this project.

General Instructions

General information about filling in the questionnaire: [Click Here]

As we mentioned this questionnaire will take approximately 30-40 minutes to complete. If you are unable to complete this questionnaire by yourself you can ask a carer or family member to assist you or one of our research nurses can help you. Please let us know who will be filling in this questionnaire?:

- (●) I am filling this in myself
- ( ) I will have help from a carer or family member
- ( ) The research nurse is assisting me

Informed Consent

I have read the Participant Information Sheet or someone has read it to me in a language that I understand. I agree to participate and provide information about myself as required.

- (●) I agree
- ( ) I disagree and don't want to participate in this survey

Demographics

We would like to ask you some personal questions about your name, date of birth and gender so we can ensure that we accurately match the information you provide to us in this questionnaire with your clinical information.

What is your first name?

Test

What is your surname?

Test

What is your gender?

♀ Female | ♂ Male

What is your date of birth?

13 / Mar / 1992

Biometrics

What is your height (m)?

2

What is your current weight (kg)?

62

Your current **BMI** is 15.5

Current Residential Information

The following questions will ask you about the places you have lived during different periods of your life. We would like to know the postcode of the area in which you lived however if you don't know the postcode, please just write the name of the place.

If you lived overseas during any of the specified periods of your life, please fill in the name of the place and the country.

What is the Australian postcode of your current residence?
Find a postcode here: http://auspost.com.au/postcode

What is the name of the town/city in which you currently reside?

Which **major** town/city is closest to where you currently reside?
e.g., State capital; capital city

Do you currently live on a farm or in a town dwelling?

- ○ Farm
- ○ Town dwelling
- ● Leave blank

Residential History

Did you live in Australia or overseas for the majority of the following periods of your life?

	Australia	Overseas	Leave blank
Age 0-12	●	○	○
Age 13-20	●	○	○
Age 21-45	○	●	○
Age 46+	○	●	○

Where did you live for the majority of your life aged 0-12? (Australian Postcode of the town/city in which you resided, leave blank if unknown)
Find a postcode here: http://auspost.com.au/postcode

Where did you live for the majority of your life aged 0-12? (Name of the town/city in which you resided)

Where did you live for the majority of your life aged 13-20? (Australian Postcode of the town/city in which you resided, leave blank if unknown)
Find a postcode here: http://auspost.com.au/postcode

Where did you live for the majority of your life aged 13-20? (Name of the town/city in which you resided)

Where did you live for the majority of your life aged 21-45? (Placename and country, e.g.: London, United Kingdom)

Where did you live for the majority of your life aged 45+? (Placename and country, e.g.: London, United Kingdom)

Education & Occupation

We would like to ask you some questions about your education and the occupations you have had during your life. If you have had more than one occupation or have moved locations then please fill in one section for each different occupation you have had.

What is your highest level of completed education/qualification?

- ○ Primary School
- ○ Year 10 (or equivalent)
- ○ Year 12 (or equivalent)
- ○ TAFE/trade qualification (or equivalent)
- ○ Certificate/diploma
- ○ Undergraduate
- ○ Postgraduate
- ● Leave blank

What describes your current working status?

- ○ Full-time
- ○ Part-time
- ○ Unemployed - seeking work
- ○ Not in the labor force/retired/pensioner
- ○ Student
- ○ Other (please specify)
- ● No answer

Please enter your comment here:

What is your current occupation or, if retired/unemployed, what was your last occupation?

- ○ **Manager, Legislator or Senior Official**
- ○ **Professional**
  e.g., Engineer; IT; medical doctor; teacher; accountant; lawyer; psychologist; librarian; other professional
- ○ **Technician and Associate Professional**
  e.g., engineer technician; lab technician; computer assistant; ship and aircraft controller or pilot
- ○ **Clerical Support Worker,**
  e.g., office clerk; customer services clerk; secretary; cashier; travel agency clerk; receptionist
- ○ **Services Worker and Shop and Market Sales Worker**
  e.g., travel guide; housekeeper; waiters; waitress or bartender
- ○ **Skilled Agricultural, Forestry and Fishery worker**
  e.g., market-oriented skilled agricultural or fishery worker
- ○ **Craft and related Trades Worker**
  e.g., extraction (mining) and building trades worker; metals worker; machinery trades worker; precision; welder
- ○ **Plant and Machine Operator or Assembler**
  e.g., stationary-plant or related-operator; machine operator or assembler
- ○ **Elementary Occupation**
  e.g., homemaker; street vendor; domestic helper, cleaner or launderer; garbage collector; agricultural, fishery; truck driver; bus/train driver
- ○ **Armed Forces Occupation**
- ○ **Other**
  please specify
- ● No answer

Please enter your comment here:

Please enter the Australian postcode of your current or last place of work (Leave blank if unknown or overseas)
Find a postcode here: http://auspost.com.au/postcode

Please enter the name of the town/city and country where your current or last place of work is located (e.g.: London, United Kingdom or Brisbane, Australia)

Did you hold any other occupation for more than 5 years before your current/last occupation?

Yes | No | Leave

		blank

What type of occupation was this?

- ◯ **Manager, Legislator or Senior Official**
- ◯ **Professional**
  e.g., Engineer; IT; medical doctor; teacher; accountant; lawyer; psychologist; librarian; other professional
- ◯ **Technician and Associate Professional**
  e.g., engineer technician; lab technician; computer assistant; ship and aircraft controller or pilot
- ◯ **Clerical Support Worker,**
  e.g., office clerk; customer services clerk; secretary; cashier; travel agency clerk; receptionist
- ◯ **Services Worker and Shop and Market Sales Worker**
  e.g., travel guide; housekeeper; waiters; waitress or bartender
- ◯ **Skilled Agricultural, Forestry and Fishery worker**
  e.g., market-oriented skilled agricultural or fishery worker
- ◯ **Craft and related Trades Worker**
  e.g., extraction (mining) and building trades worker; metals worker; machinery trades worker; precision; welder
- ◯ **Plant and Machine Operator or Assembler**
  e.g., stationary-plant or related-operator; machine operator or assembler
- ◯ **Elementary Occupation**
  e.g., homemaker; street vendor; domestic helper, cleaner or launderer; garbage collector; agricultural, fishery; truck driver, bus/train driver
- ◯ **Armed Forces Occupation**
- ◯ **Other**
  please specify
- ◉ No answer

Please enter your comment here:

Please enter the Australian postcode of your place of work during that occupation (Leave blank if unknown or overseas)
Find a postcode here: http://auspost.com.au/postcode

What is the name of the town/city and country where your place of work was located during that occupation (e.g.: London, United Kingdom or Brisbane, Australia)?

Did you hold any other occupation for more than 5 years before your last mentioned occupation?

Yes	No	Leave blank

Have you ever been in the military? (conscripted/voluntarily)

Yes | No | Leave blank

Which armed forces did you serve?

- ( ) Army
- ( ) Navy
- ( ) Airforce
- ( ) Special Units
- ( ) Other (please specify)
- (•) No answer

Please enter your comment here:

Have you ever been deployed for a period of more than one month?

Yes | No | Leave blank

Where have you been deployed?

Have you ever worked on a submarine?

Yes | No | Leave blank

For what length of time have you worked on a submarine?

Family History

In this section we would like to ask you some questions about your immediate family including any children you may have.

For the purposes of this survey we would like to know specifically about your biological family members. For this reason, we would like to ask you about your relationship to your family members and whether your biological family members are known to you.

A biological family member is someone that is directly related to you by blood, for example your natural mother or father, full or half sibling, or natural child.

A non-biological family member is not directly related to you by blood, for example adoptive parents, step parents, step siblings, adopted siblings, adoptive children, and step children.

We will ask you about the biological family that is known to you, their relationship to you, and whether they are still living. Please answer as best you can.

Are your natural parents known to you?

For the purposes of the survey please choose yes if you know who they are, even if they are no longer living or are estranged.

- (●) Yes, both
- ( ) Yes, mother
- ( ) Yes, father
- ( ) Leave blank

Is your father still living?

Yes	No	Leave blank

What is your father's current age?

Is your mother still living?

Yes	No	Leave blank

How old was your mother when she died?

What was the cause of your mother's death?

Do you have any biological siblings? i.e. full or half brothers or sisters

- (●) Yes
- ( ) No
- ( ) Leave blank

How many biological siblings do you have?

2

Please complete the following table with information regarding your siblings. Take number one as the first-born.

	Brother/Sister (half/full)	Still Living?	Current age/ Age when deceased *Please state months or years*	Cause of death *(if applicable)*
1	Brothe	No		
2	Sister (	Yes		

Have you had any biological children?

- (●) Yes
- ( ) No
- ( ) Leave blank

How many children have you had? (0 if none)

2

Please complete the following table regarding your children. Take number one as the first-born child.

	Gender	Still living?	Current age/ Age when deceased *Please state months or years*	Cause of death *(if applicable)*
1	Male	Yes		
2	Fema	No		

Ancestry

Which ancestral group(s) do you most closely relate to **biologically**? (multiple answers possible)

- [ ] Oceanian (Australian Peoples, New Zealand Peoples, Melanesian and Papuan, Micronesian, Polynesian)
- [ ] Aboriginal & Torres Strait Islander
- [ ] European (North-West European, Southern European, Eastern European)
- [ ] African (includes African American or Afro-Caribbean)
- [x] Middle Eastern
- [ ] East Asian (from China, Japan, Korea, South-East Asia)
- [ ] South Asian (from India, Pakistan, Bangladesh, Sri Lanka)
- [x] South American
- [ ] Other:

Which ancestral group(s) does your **natural mother** most closely relate to biologically? (multiple answers possible)

- [ ] Oceanian (Australian Peoples, New Zealand Peoples, Melanesian and Papuan, Micronesian, Polynesian)
- [x] Aboriginal & Torres Strait Islander
- [x] European (North-West European, Southern European, Eastern European)
- [ ] African (includes African American or Afro-Caribbean)
- [ ] Middle Eastern
- [ ] East Asian (from China, Japan, Korea, South-East Asia)
- [ ] South Asian (from India, Pakistan, Bangladesh, Sri Lanka)
- [ ] South American
- [ ] Other:

Which ancestral group(s) does your **natural father** most closely relate to biologically? (multiple answers possible)

- [ ] Oceanian (Australian Peoples, New Zealand Peoples, Melanesian and Papuan, Micronesian, Polynesian)
- [x] Aboriginal & Torres Strait Islander
- [ ] European (North-West European, Southern European, Eastern European)
- [ ] African (includes African American or Afro-Caribbean)
- [ ] Middle Eastern
- [ ] East Asian (from China, Japan, Korea, South-East Asia)
- [ ] South Asian (from India, Pakistan, Bangladesh, Sri Lanka)
- [ ] South American
- [ ] Other:

Diagnosis

Do you have a current MND diagnosis?

Yes | No

We would like to ask you some questions about your MND diagnosis. Please fill in this section as best you can.

What was the date of your MND diagnosis?

18 / Jun / 2010

What type of MND do you have?

- ( ) Familial
- ( ) Sporadic
- (•) Leave blank

Are you currently taking any medication for MND?

Yes | No | Leave

blank

Please list the medication you are currently taking for MND

Medical History

This section of questions will ask you about any other medical conditions or disorders you may have had in your lifetime. This information may be able to assist us in looking at other medical conditions that may influence the onset of MND or other neurological disorders. If you have had one of these conditions or disorders you may be asked additional questions about this.

Have you ever experienced, been diagnosed or treated for any medical condition in the groups listed below? Please fill in the table.

	Have you ever had a disease that can be subdivided into one of the following categories?	How old were you when this occurred?	What did you have?
**Infectious and parasitic diseases** *e.g.: hepatitis, STDs, tuberculosis, herpes, mosquito-related diseases, tick-related diseases , influenza, viral or bacterial infections*	Yes		
**Cancers/Neoplasms**	Yes		
**Diseases of the blood and blood-forming organs and disorders involving the immune mechanism** *e.g.: anaemias*	Yes		
**Endocrine, nutritional and metabolic diseases** *e.g.: thyroid gland, diabetes, endocrine glands, malnutrition, obesity*	Yes		
**Mental and behavioural disorders** *e.g.: depression, schizophrenia, bipolar, anxiety*	Yes		
**Diseases of the nervous system** *e.g.: inflammatory diseases, Parkinson's, Alzheimer's*	Yes		
**Diseases of the eye and adnexa**	Please choose...		
**Diseases of the ear and mastoid process**	Please choose...		
**Diseases of the circulatory system** *e.g.: rheumatic, hypertensive, ischaemic heart diseases, diseases of arteries/veins/lymphatic vessels/nodes*	Yes		
	Please choose...		

**Diseases of the respiratory system**<br>*e.g.: lung diseases, respiratory tract diseases*			
**Diseases of the digestive system**<br>*e.g.: intestines, liver, pancreas, stomach, hernia*	Please choose...		
**Diseases of the skin and subcutaneous tissue**	Please choose...		
**Diseases of musculoskeletal system and connective tissue**<br>*e.g.: diseases of muscles/joints*	Please choose...		
**Diseases of the genitourinary system**<br>*e.g.: kidney, urinary system, breasts, genital organs*	Please choose...		
**Congenital malformations, deformations and chromosomal abnormalities**<br>*e.g.: spina bifida, encephalocele, down's syndrome*	Please choose...		

Did you ever have serious trauma requiring medical care?

Yes | No | Leave blank

Please fill in the following table regarding trauma. Use one row of information for each trauma event.
Please enter numbers only (except for 'other' specification)

	**Injury Type**<br>*1: Head injury w/ concussion*<br>*2: Fracture*<br>*3. Contusion*<br>*4: Sprain*<br>*5: Strain*<br>*6: Other (Specify)*	**Age at Occurrence**	**Circumstances**<br>*1: Work*<br>*2: Sport*<br>*3: Leisure (other than sport)*<br>*4: Traffic*<br>*5: Other (Specify)*	**Injury Duration**<br>*1: Temporary*<br>*2: Permanent Injury*	**Injured Body Part**<br>*1: Head*<br>*2: Arm(s)*<br>*3: Chest*<br>*4: Abdomen*<br>*5: Legs(s)*<br>*6: Spine*<br>*7: Other (Specify)*	**Severity**<br>*1: Mild*<br>*2: Moderate*<br>*3: Severe*
1	1	45	2	1	6	4
2						
3						
4						
5						
6						
7						
8						

You have reported in the 'infectious and parasitic diseases' question of the medical history section. Have you had any of the following bacterial infections within the last 12 months?

- [ ] Tetanus
- [ ] Whooping cough
- [ ] Streptococcal infections
- [ ] Bacterial meningitis
- [ ] Legionnaire's Disease
- [ ] Q Fever
- [ ] Diptheria
- [ ] Listeria
- [ ] Sepsis
- [ ] Other infection not included in this list
- [ ] No

Have you had any of the following parasitic or viral infections within the last 12 months?

- [ ] Influenza
- [ ] Lyme disease
- [ ] Viral meningitis
- [ ] Shingles
- [ ] Dengue Fever
- [ ] Malaria
- [ ] Gastroenteritis
- [ ] Herpes simplex 1 (HSV-1) - cold sore virus
- [ ] Other infection not included in this list
- [ ] No

Have you have one of the following sexually transmitted infections (STI, formerly known as STD) within the last 12 months?

- [ ] Chancroid
- [ ] Chlamydia
- [ ] Gonorrhoea
- [ ] Hepatitis
- [ ] Herpes simplex (HSV-2)
- [ ] HIV
- [ ] Human Papillomavirus (HPV)
- [ ] Syphilis
- [ ] Trichomoniasis
- [ ] Other STD not on this list
- [ ] No

What kind of cancer treatment(s) did you receive (multiple answers possible)

- [ ] Radiation Therapy
- [ ] Surgery

- [ ] Chemotherapy
- [ ] Other:

Were you diagnosed with Parkinson's Disease within the last 12 months?

Yes | No | Leave blank

Were you diagnosed with Alzheimer's Disease within the last 12 months?

Yes | No | Leave blank

Were you, besides Alzheimer's or Parkinson's, diagnosed with any other long-term brain disease within the last 12 months?

Yes | No | Leave blank

Have you had any major surgery within the last 12 months?

Yes | No | Leave blank

Please specify what major surgery/surgeries you had within the last 12 months

Were you diagnosed with any movement disorder within the last 12 months?

Yes | No | Leave blank

Were you diagnosed with depression within the last 12 months?

Yes | No | Leave blank

Were you diagnosed with any bipolar disorder within the last 12 months?

Yes | No | Leave blank

Were you diagnosed with any anxiety disorder within the last 12 months?

Yes | No | **Leave blank**

Have you been diagnosed with any other mental disorder or brain disease not mentioned above within the last 12 months?

Yes | No | Leave blank

Please specify which other mental disorder or brain disease you were diagnosed with, within the last 12 months?

Were you treated for high cholestrol levels within the last 12 months?

Yes | No | **Leave blank**

Were you treated for high blood pressure within the last 12 months?

Yes | No | **Leave blank**

Medication History

We would like to ask you some questions about medications you have been prescribed for diagnosed medical conditions or disorders that you have told us about. Please fill in each section as best you can.

Did you have all **childhood** vaccinations according to the vaccination program of your country?

Yes | **No** | Leave blank

Which vaccinations **did** you receive?

- [ ] Hepatitis B
- [ ] Diptheria
- [ ] Tetanus
- [ ] Whooping cough
- [ ] Influenza
- [ ] Polio

- [ ] Rotavirus
- [ ] Pneumococcal conjugate
- [ ] Measles, Mumps, Rubella and Varicella (chickenpox)
- [ ] None
- [ ] Other: ____

Did you use medication for high cholesterol within the last 12 months?

Yes | No | Leave blank

What medication did you use for high cholesterol in that period and what year did you start taking it?

____

Did you use medication for high blood pressure within the last 12 months?

Yes | No | Leave blank

What medication did you use for high blood pressure in that period and what year did you start taking it?

____

Have you ever been prescribed medication for anxiety or depression?

Yes | No | Leave blank

Please fill in the following table regarding medication prescribed for anxiety or depression. Please include age started and stopped
Leave 'Age Stopped' blank if you are still using it

	Used: Yes/No	Age Started	Age Stopped *(if applicable)*
**Diazepam** *Valium*	Please choose...		
**Duloxetine** *Cymbalta*	Please choose...		
**Venlafaxine** *Efexor*	Please choose...		
**Escitalopram** *Lexapro, Zoloft*	Please choose...		
**Sertraline** *Lustral, Zoloft*	Please choose...		
**Fluoxetine** *Prozac*	Please choose...		
**Citalopram** *Cipramil*	Please choose...		
	Please choose...		

**Sodium Valproate** *Epilim*			
**Lamotrigine** *Lamictal*	Please choose...		
**Lofepramine** *Gamanil*	Please choose...		
**Mirtazepine** *Zispin, Avanza*	Please choose...		
**Trazodone**	Please choose...		
**Paroxetine** *Seroxat, Aropax*	Please choose...		
**Lithium** *Priadel*	Please choose...		
**Dothiepin** *Prothiaden, Dothem*	Please choose...		
**Trimipramine** *Surmontil*	Please choose...		
**Buproprion** *Zyban*	Please choose...		

Have you ever been prescribed anti-psychotics?

Yes | No | Leave blank

Please fill in the following table regarding anti-psychotics. Also, include age started and stopped
Leave 'Age Stopped' blank if you are still using it

	**Used: Yes/No**	**Age Started**	**Age Stopped** *(if applicable)*
**Trifluoperazine** *Stelazine*	Please choose...		
**Arpiprazole** *abilify*	Please choose...		
**Chlorpromazine** *Largactil*	Please choose...		
**Clozapine** *Clozaril*	Please choose...		
**Flupenthizol** *Depixol*	Please choose...		
**Sulpiride** *Dolmatil*	Please choose...		
**Ziprasidone** *Geodon, Zeldox*	Please choose...		
**Haloperidol** *Haldol*	Please choose...		
**Fluphenazine**	Please choose...		
**Risperidone**	Please choose...		
**Quetiapine** *Seroquel*	Please choose...		
**Olanzepine** *Zyprexa*	Please choose...		

**Thioridazine** *Melleril*	Please choose...		

Did you take any other medication within 12 months prior to the onset of your MND symptoms?

Yes | No | Leave blank

Please fill in the following table regarding medication used within 12 months prior to the onset of your MND symptoms

	**Name of the Drug**	**Reason**	**Age Started**	**Age stopped** *(if applicable)*
1				
2				
3				
4				
5				
6				
7				
8				

Family Medical History

Do you know of any blood relative of yours that has been diagnosed with MND?

Yes | No | Leave blank

How is/are this/these person(s) related to you?

- ☐ Parent
- ☐ Sibling
- ☐ Grandfather/Grandmother
- ☐ Aunt/Uncle
- ☐ Nephew/Niece
- ☐ Other: ______

Menstruation & Pregnancy

This section asks specifically about your health in relation to fertility and any pregnancies you may have had. If you feel uncomfortable answering any of the questions, just a reminder you don't need to answer any questions you don't want to, just leave them blank.

At what age did you have your first period?

[ ]

Has there ever been a time when your cycle was irregular for more than 3 months?

Yes | No | Leave blank

Around what age was your cycle irregular?

- ○ Age 0-12
- ○ Age 13-20
- ○ Age 21-45
- ○ Age 46+
- ● Leave blank

Are you currently using hormonal contraceptives or have you ever done so?

Yes | No | Leave blank

In what form have you used hormonal contraceptives?

- ☐ Pill
- ☐ Subcutaneous implant
- ☐ Injection
- ☐ Other: [ ]

At what age did you start using hormonal contraceptives?

[ ]

For how many years did you/have you been using hormonal contraceptives?

[ ]

If you have ever fallen pregnant, how many times have you been pregnant?

2

Did you breastfeed any of your children for at least 6 weeks after birth?

Yes | No | Leave blank

On average, how many months did you breastfeed your child/children?

- ( ) Less than 1 month
- ( ) 1-2 months
- ( ) 3-5 months
- ( ) More than 5 months
- (•) Leave blank

Have you experienced infertility?

Yes | No | Leave blank

Have you ever been diagnosed with one of the following diseases?

- [ ] Fibroids
- [ ] Polycystic Ovary Syndrome
- [ ] Endometriosis
- [ ] None of the above

Have you had a hysterectomy? (removal of the uterus)

Yes | No | Leave blank

At what age did you have a hysterectomy?

Were your ovaries removed?

- (•) Yes, both
- ( ) Yes, on one side
- ( ) No
- ( ) Leave blank

At what age were your ovaries removed?

Have you started menopause?

Yes | No | Leave blank

At what age did you start menopause?

Lifestyle Section A

We would like to ask you some questions about your lifestyle and behaviours during your lifetime. These questions are about your alcohol and smoking habits as well as questions about performance-enhancing supplements and recreational drugs. Scientific research to date shows us that consumption of these substances can influence the onset of MND and other neurological conditions and so we feel these questions are important to ask. These questions are not compulsory and if you feel uncomfortable answering them, please just leave them blank.

Do you drink alcohol or have you ever done so?

- ○ I drink alcohol regularly
- ○ I drink alcohol occasionally
- ● I no longer drink alcohol
- ○ I have never drunk alcohol
- ○ Leave blank

How often do you drink alcohol?

- ○ Daily or almost daily
- ● Weekly
- ○ Monthly
- ○ Once / couple of times (just to try it)
- ○ Given up drinking
- ○ Never
- ○ Leave blank

At what age did you start drinking alcohol?

At what age did you stop drinking alcohol?

How many standard glasses of alcohol do you or did you drink on average **per week** when you were using it **the most**?

1 standard drink equals:

Beer (4.8%) 270ml

Wine (13%): 100ml

Spirits (40%): 30 ml

Has there been a period where you abstained from drinking alcohol for at least 3 months?

Yes | No | Leave blank

How many times did you stop drinking alcohol for more than 3 months?

What was the longest period you stopped drinking alcohol? Please specify if months or years

Lifestyle Section B

Have you ever used drugs? (e.g.: cocaine, stimulants, inhalants, sedatives, hallucinogens, opioids, party drugs)

Yes | No | Leave blank

During your lifetime, which of the following substances have you ever used?

This **DOES NOT** include drugs that were prescribed to you for health reasons and which you took according to the directions.

	Have you ever used this drug?	How often did you use it, when you were using it the most?	At what age did you first use it?
**Cocaine** *e.g.: coke, crack*	Please choose...	Please choose...	
**Cannabis** *marijuana, skunk, hash, pot*	Yes	Please choose...	
**Amphetamine-type stimulants** *e.g.: speed, ice, diet pills*	Please choose...	Please choose...	
**Inhalants** *e.g.: nitrous, glue, petrol, paint thinner*	Please choose...	Please choose...	
**Sedatives or sleeping pills** *e.g.: valium, serepax, rohypnol*	Please choose...	Please choose...	
**Hallucinogens** *e.g.: LSD, acid, mushrooms, PCP*	Please choose...	Please choose...	

**Opioids** *e.g.: heroine, morphine, fentanyl, methadone, codeine*	Please choose...	Please choose...	
**Ecstasy, ketamine, GHB or party drugs** *e.g.: E, X, MDMA, K, special K, Fantasy*	Please choose...	Please choose...	

Have you ever used any other drug not mentioned in the previous table?

Yes | No | Leave blank

Please describe which drugs(s) you have used other than those already mentioned above

	Name of the drug	At what age did you first use it?	When you were using it the most, how often did you use it?
1			Please choose...
2			Please choose...
3			Please choose...
4			Please choose...
5			Please choose...

Have you ever used drugs in combination with each other? (including alcohol and cannabis)

Yes | No | Leave blank

Which combinations did you use the most often? *(e.g.: Alcohol & Cocaine or Cannabis & LSD)*

Combination 1

Combination 2

Combination 3

Combination 4

Combination 5

Combination 6

Combination 7

Combination 8

Lifestyle Section C

Did you ever use performance-enhancing drugs (oral) (e.g.: creatine, steroids, amphetamines, adrenaline, oxycodone, methadone etc)

Yes | No | Leave blank

What type(s) did you use and at what age did you start/stop using it?

	Used: Yes/No	Age Started	Age Stopped *(if applicable)*
Creatine	Please choose...		
Anabolic Androgenic Steroids	Please choose...		
Clenbuterol, tibolone, zeranol, zilpaterol	Please choose...		
Amphetamines	Please choose...		
Adrenaline	Please choose...		
Heroin, fentanyl hydromorphine/hydromorfine, methadone, morphine, oxycodone, oxymorphone/oxymorfine, pentazocine, pethidine	Please choose...		

Have you ever used Intramuscular Performance Enhancing Drugs?

Yes | No | Leave blank

What type(s) did you use and at what age did you start/stop using it?

	Used: Yes/No	Age Started	Age Stopped *(if applicable)*
Erythropoietin (EPO), dEPO, CERA or hematide	Please choose...		
Chorionic Gonadotrophin (CG)	Please choose...		
Growth Hormone (GH)	Please choose...		
Luteinizing Hormone (LH)	Please choose...		
Insulin-like Growth Factor-1 (IGF-1)	Please choose...		
Mechano Growth Factors (MGFs)	Please choose...		
Platelet-Derived Growth Factors (PDGF)	Please choose...		

Fibroblast Growth Factors (FGFs)	Please choose...		
Vascular Endothelial Growth Factor (VEGF)	Please choose...		
Hepatocyte Growth Factor (HGF)	Please choose...		

Lifestyle Section D

What is your current smoking status?

- ○ Current smoker (daily or almost daily)
- ○ Occasional smoker
- ● Quit smoking
- ○ Never smoked
- ○ Leave blank

What forms of tobacco have you used?

- ☐ Cigarettes (including self-rolled)
- ☐ Cigars
- ☐ Pipe
- ☐ Other (e.g.: menthol cigarettes, e-cigarettes, bidis, hookahs and shisha) (Please Specify)

At what age did you smoke for the first time?

When you were smoking the heaviest how many cigarettes would you smoke in a day?

At what age did you stop smoking?

Has there been a period where you abstained from smoking for more than 3 months?

Yes | No | Leave blank

What was the longest period you stopped smoking for? Please specify if months or years
e.g., 3M for 3 months, 1Y for 1 year

Lifestyle Section E

You told us in Lifestyle Section B that you have used cannabis. We would like to ask you some questions about your cannabis use.

At what age did you first use cannabis?

When you were using cannabis the most, how often did you use it?

- ○ Daily or almost daily
- ○ Weekly
- ○ Monthly
- ○ Once/few times (just to try)
- ● Leave blank

Physical Activity

Physical activity is an important part of maintaining good general health. We know that regular exercise has beneficial effects for overall wellbeing and in some cases can reduce the onset or development of some conditions and disorders. In this section we will ask you some questions about how much physical activity you have had during periods of your lifetime.

Please describe your overall experience with physical activity during certain periods of your life below

	**Light** *e.g.: slow walking, gentle swim, childcare*	**Moderate** *e.g.: football, tennis, jogging, golf, gardening, house cleaning, brisk walk*	**Strenuous** *e.g.: running, strength training, HIIT, road cycling, track and field, ball sports*	No answer
**13-20 years old**	○	○	○	●
**20-45 years old**	○	○	○	●
**5 years before onset of symptoms** *(if applicable)*	○	○	○	●
**Last 10 years**	○	○	○	●

Have you ever played on a grass sports field for more than 10 hours/week for more than 2 consecutive years? (approximately)

Yes | No | **Leave blank**

Diet

*A balanced diet provides your body with the vital nutrients it needs to build and maintain healthy cells, tissues and organs, which are crucial for maintaining good general health. This section asks you to provide information on your current diet. Think about your diet over the **LAST MONTH**, including supplements. (We have displayed the Australian recommended diet pyramid to help you remember. Just answer as best you can)*

Do you adhere to any specific dietary practices?

- ☐ Organic
- ☐ Vegetarian
- ☐ Vegan
- ☐ Lactose Free
- ☐ Gluten Free
- ☐ I don't have any specific dietary practice
- ☐ Other: ____________

Using the advised food intake as seen in the food pyramid below, how many servings of each food group would you have on an average day?

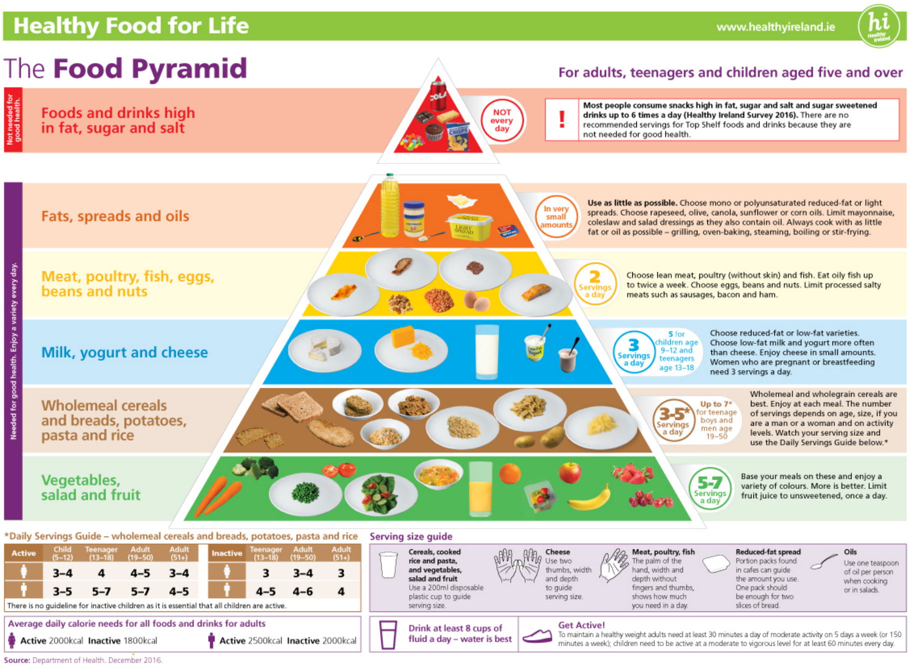

Source: http://www.safefood.eu/Healthy-Eating/What-is-a-balanced-diet/The-Food-Pyramid.aspx

	Average servings per day (0-10)
**Foods and drinks high in fat, sugar and salt**<br>*Max. once/twice a week*	0
**Fats, spreads and oils**<br>*Daily in very small amounts*	0
**Meat, poultry, fish, eggs, beans and nuts**<br>*2 servings a day*	0

Milk, yogurt and cheese
3 servings a day

0

Wholemeal cereals and breads, potatoes, pasta and rice
3-5 servings a day, up to 7 for men aged 12-50

0

Vegetables, salad and fruit
5-7 servings a day

0

Did you take any dietary supplements (vitamins, minerals, fish oil etc.) before you were diagnosed with MND?

Yes | No | Leave blank

Which types of dietary supplements did you take before you were diagnosed with MND?

- [ ] Fish oil
- [ ] Multivitamins
- [ ] Vitamin D
- [ ] CoQ10
- [ ] Vitamin B
- [ ] Calcium
- [ ] Magnesium
- [ ] Probiotics
- [ ] Vitamin C
- [ ] Glucosamine
- [ ] Other:

How often would you take these dietary supplements?

- ○ Multiple times a day
- ○ Once a day
- ○ Multiple times a week
- ○ Once a week or less
- ● Leave blank

Do you currently take any dietary supplements (vitamins, minerals, fish oil etc.)?

Yes | No | Leave blank

Which dietary supplements do you currently take?

- [ ] Fish oil
- [ ] Multivitamins
- [ ] Vitamin D
- [ ] CoQ10
- [ ] Vitamin B

- [ ] Calcium
- [ ] Magnesium
- [ ] Probiotics
- [ ] Vitamin C
- [ ] Glucosamine
- [ ] Other: ______

How often do you take these dietary supplements?

- ○ Multiple times a day
- ○ Once a day
- ○ Multiple times a week
- ○ Once a week or less
- ● Leave blank

How many calories does your diet usually contain?

Within a healthy, balanced diet, a man needs around ***2,500 calories*** *a day to maintain his weight. For a woman, that figure is around* ***2,000 calories*** *a day.*

- ○ Less than the advised amount of calories
- ○ Around the advised amount of calories
- ○ More than the advised amount of calories
- ● Leave blank

On average men need ***300g*** *of carbohydrates per day while women need around* ***230g****.*

How much carbohydrate does your daily diet usually contain?

Examples of carbohydate amounts in specific foods: 1 cup of pasta/rice (45g), 1 medium potato (30g), 1 cup of corn or peas (30g), 1 cup of veggies (15g), small fruit (15g), 1 cup of milk/yogurt (12g), 1 slice of bread (15g)

- ○ Less than 150 grams
- ○ 150-250 grams
- ○ 250-400 grams
- ○ 400-600 grams
- ○ 600+ grams
- ● Leave blank

Did you change your diet after your MND diagnosis?

[Yes] [No] [Leave blank]

In which way did you change your diet?

- [ ] More calories
- [ ] Less calories
- [ ] More supplements
- [ ] Less supplements
- [ ] More carbohydrates

☐ Less carbohydrates

☐ Other:

How often do you eat fish or shellfish?

- ○ Daily
- ○ 4-6 times a week
- ○ 2-3 times a week
- ○ Once a week
- ○ 2-3 times a month
- ○ Once a month or less
- ○ Never
- ● Leave blank

Do you have a drink containing caffeine daily or almost daily?

Yes | No | Leave blank

How many drinks containing caffeine do you have on a typical day?

- ○ 1-2 drinks
- ○ 3-4 drinks
- ○ 4-5 drinks
- ○ 6-9 drinks
- ○ More than 10 drinks
- ● Leave blank

Environmental Factors

Have you ever used or been excessively exposed to one of the following chemical hazards:

	**Exposed: Yes/No/Uncertain**	**Type(s)**	**Period/Duration** *Enter in hrs/day and days/year format*
**Animal and plant toxins** *e.g.: venom or poison, exposure through bites, stings etc.*	Please choo:		
**Drugs and pharma-ceuticals** *e.g.: used in treat-ment of livestock*	Please choo:		
**Endocrine disrup-tors** *e.g.: natural hor-mones, natural chem-icals, synthetically produced pharma-ceuticals, man-made chemicals*	Please choo:		
	Please choo:		

**Neurotoxins** *e.g.: Botulinum toxin, Polybrominated Diphenyl Ethers (PBDEs), Isobutyronitrile, Hexachlorophene, Metaldehyde, Propoxur, Hexane, Styrene, Bifenthrin*			
**Pesticides** *e.g.: fungicides, insecticides, herbicides, rodenticides, antimicrobials*	Please choos		
**Persistent Environmental contaminants** *e.g.: Dioxins (TCDD & Furans, Benzo(a)pyrene, Octachlorostyrene, Pentabromo diphenyl ether (PBDEs), Polybrominated hydrocarbons, Polychlorinated biphenyls (PCBs), Polycyclic aromatic hydrocarbons (PAHs, Tin)*	Please choos		
**Solvents** *e.g.: Acetone, Ethyl Alcohol, Benzene, Carbon Disulphide, Chloroform, 1,3-Dichloropropane, Ethyl Acetate, Methyl Cellosolve, Nitrobenzene, 2-Nitropropane, Pyridine, Trichloroethylene, Vinyl Cloride, Xylene*	Please choos		
**Acids** *e.g.: Sulphuric, Nitric, Hydrochloric, Citric, Acetic*	Please choos		

Have you ever used or been excessively exposed to one of the following physical hazards:

	Exposed: Yes/No/Uncertain	Type(s)	Period/Duration
**Heavy metals** *e.g.: Arsenic, Cadmium, Lead, Magnesium, Mercury, Plutonium, Thallium, Uranium*	Please choos		
**Electricity / Elecotromagnetic Fields**	Please choos		
**Radiation** *e.g.: UV, X-ray, Alpha, Beta, Gamma (Nuclear), Neutron, Microwave*	Please choos		

Did you ever endure an accidental electric shock?
Do Not include Electroconvulsive therapy - ECT

Yes | No | Leave blank

On average, how many hours do you spend outside per week?

- ( ) More than 25 hours
- ( ) 20-25 hours
- ( ) 15-20 hours
- ( ) 10-15 hours
- ( ) 5-10 hours
- ( ) Less than 5 hours
- (•) Leave blank

In which situation(s) would you put on sunscreen on a single day? (Multiple answers possible)

- [ ] Always
- [ ] Full day in swimwear outside
- [ ] 2 hours in swimwear outside
- [ ] Full day of hiking
- [ ] 2 hours cycling tour
- [ ] 30 minute walk
- [ ] Never

Have you ever been, or are you, exposed to diesel fuel or fumes on a regular basis (i.e. at least once a week for 6 months or more), in any of the ways listed below? (multiple answers possible)

- [ ] Living near a major road or highway
- [ ] Living in an inner-city
- [ ] Commuting to and from work for a daily total of 2 hours or more
- [ ] Driving a diesel-fuelled passenger vehicle
- [ ] Driving a diesel fuelled light commercial vehicle
- [ ] Driving a bus
- [ ] Driving a truck
- [ ] Driving a land, sea or air military vehicle
- [ ] Using diesel-fuelled farm equipment
- [ ] Working on a diesel-fuelled boat or ship
- [ ] Operating heavy machinery
- [ ] Working on or at a railroad, mine, toll-booth, dock or garage/petrol station
- [ ] Traveling on a school bus as a child
- [ ] Cooking on a diesel-fuelled stove
- [ ] Using a diesel-fuelled heater
- [ ] Other: ____

Your Personal Journey

We have created this section for you to fill in with any additional information that you think may be of relevance to your MND diagnosis.

It could be information about environmental exposures you have experienced that we have not asked about in this questionnaire, or information about events in your life that may have impacted on your diagnosis. You can also use this section to expand on any of your previous answers.

All information is important to us, so please fill this in.

Please fill in anything you would like to tell us in the text box below

MND RIA

Are you receiving services and/or support from your state MND association?

Yes | No | Leave blank

Please choose the services and/or support you are receiving from your state MND association (you can choose more than one)

- [ ] Information
- [ ] Ongoing support
- [ ] Home visits
- [ ] Assistance in accessing MND Clinics and local services
- [ ] Equipment or assistance in accessing equipment from government agencies
- [ ] Peer support and education programs
- [ ] Carer workshops and programs

☐ Support groups

☐ Education sessions and information for health and community care providers

☐ Other:

Where do you prefer to get information about MND? You can choose more than one.

☐ Internet

☐ MND association

☐ Your neurologist

☐ Other health care provider

☐ MND support group

☐ Webinar

☐ Information session

☐ Electronic newsletter

☐ Paper newsletter

☐ Application (app) on mobile device

☐ Other:

What do you think is missing (if anything) from the information about MND available from the MND associations in Australia?

○ What MND is

○ Managing and living with MND

○ Access to equipment

○ The NDIS

○ Carer support

○ Comments (please specify)

◉ No answer

Please enter your comment here:

What MND research topics are you interested in? (You can choose more than one)

☐ Genetics

☐ Nerve cells

☐ Environmental exposures

☐ Mental health and ALS

☐ Clinical trials

☐ Healthcare

☐ Other:

Submit

The University of Queensland
Brisbane St Lucia, QLD 4072
+61 7 3365 1111
© 2021 The University of Queensland | CRICOS Provider No:00025B
