# Supplementary material for: Strategic Amyotrophic Lateral Sclerosis Australia–Systems Genomics Consortium (SALSA-SGC): cohort profile
Source: BMJ Open. 2026 Jun 18;16(6):e110906. doi: 10.1136/bmjopen-2025-110906 (PMC13288974; doi:10.1136/bmjopen-2025-110906)
Supplement: online supplemental material 3 [file bmjopen-16-6-s003.pdf]

SALSA-SGC Authorship Guidelines

The consensus for the consortium regarding publication attributions is as follows:

- Main papers that use the data for the purpose of ALS research includes SALSA-SGC principal investigators and the SALS-SGC banner authorship
- Any paper that uses the data for non-ALS research including method development just uses the SALSA-SGC banner authorship
- If people apply to access the data and resource, then use SALSA-SGC banner and the SALSA-SGC PI(s) directly associated with the work will be named authorship
- Under the banner authorship, authors will be named in the supplementary material making it PubMed searchable

SALSA-SGC Flexible Governance model

The consortium governance model of SALSA-SGC allows for multiple parties across multiple institutions to co-exist as an entity and can accommodate within its Terms of Reference:

- Human Research Ethics Committee Approvals
- Clinical Trial - Human Research Ethics Committee Approvals
- Collaborative Research Agreements
- Research Service Agreements
- Data and Material Transfer agreements
- Commercial Contracts
- Clinical Standard Practices
- Trading activities

While upholding:

1. Informed consent of the participants
2. Ownership of samples and data by contributing institutions
3. Commercial waivers by participants
4. Open-source requirements by funders and publishers
5. IP generated from accessing data and / or samples
6. Appropriate attribution of contributions of members at time of publishing
7. Distribution of income
