# Supplementary material for: Strategic Amyotrophic Lateral Sclerosis Australia–Systems Genomics Consortium (SALSA-SGC): cohort profile
Source: BMJ Open. 2026 Jun 18;16(6):e110906. doi: 10.1136/bmjopen-2025-110906 (PMC13288974; doi:10.1136/bmjopen-2025-110906)
Supplement: Supplementary file 8 [file bmjopen-2025-110906.reviewer_comments.pdf]

PEER REVIEW HISTORY

BMJ Open publishes all reviews undertaken for accepted manuscripts. Reviewers are asked to complete a checklist review form and are provided with free text boxes to elaborate on their assessment. These free text comments are reproduced below.

ARTICLE DETAILS

Title (Provisional)

Cohort profile: The Strategic Amyotrophic Lateral Sclerosis Australia -Systems Genomics Consortium (SALSA-SGC)

Authors

Henders, Anjali K; Ziser, Laura; Garton, Fleur; Adams, Lorel; Ernst, Karalyn; Furlong, Sarah; Heads, Judith Anne; Heggie, Susan; Krasniqi, Ruth; Bhadravathi Lokeshappa, Madhura; Mazumder, Srestha; McNamara, Elyshia; MacShane, Amanda; Mekhael, Linda; Nunn, Lorelle; Orden, Bronwen; Ryder, Julie; Thorpe, Kathryn; Toubia, Marie; Wallace, Leanne M; Wickremeratne, Dina; Windebank, Emma; Benyamin, Beben; Ngo, Shyuan; Nicholson, Garth; Pamphlett, Roger; Steyn, Frederik J.; Visscher, Peter; Willams, Kelly L; Henderson, Robert; Kiernan, Matthew; Laing, Nigel; Mathers, Susan; McCombe, Pamela; Needham, Merrilee; Rowe, Dominic; Schultz, David; Talman, Paul; Vucic, Steve; Blair, Ian P; McRae, Allan F; Wray, Naomi

---

VERSION 1 - REVIEW

**Reviewer**	**1**
**Name**	**Stavroulakis, Haris**
**Affiliation**	**Sheffield Institute for Translational Neuroscience**
**Date**	**10-Nov-2025**
**COI**	**None**

The manuscript describes the significant initiative establishing the Strategic ALS Australia Systems Genomics Consortium (SALSA-SGC). This initiative takes the form of a national prospective cohort study linking rich, longitudinal clinical data with biomarker and ‘omics data (including genetic, plasma, and serum samples) across multiple ALS clinics in five Australian states. While this is not a national register for ALS, the database is unique in matching biological samples to clinical data for 1,386 people living with ALS, plus 388 healthy controls, and 39 asymptomatic carriers, and ALS mimics.

The manuscript is concise and very well written, successfully highlighting the SALSA-SGC dataset as a high-value resource underpinned by a much needed potential to accelerate

discovery research and clinical trials in the field of ALS. The number of contributing sites, the robust and well-thought out governance framework, and crucially the longitudinal data collection element are particular strengths that guarantee the long-term scientific value of the dataset and warrant publication.

Having reviewed the manuscript here are some minor comments/suggestions that in my view would improve clarity and strengthen the manuscript further, for the authors to consider.

In the introduction it may be useful to extrapolate from the reported prevalence of ALS in Australia to estimate the total number of people living with ALS at any given time for added context and impact.

I feel a reference or threshold that classifies ALS as a "common" disease based on the approximately 0.3% lifetime risk is required. Otherwise, I would suggest perhaps rephrasing to ALS being "not infrequent" or a similar phrase.

In the cohort description, it is not immediately clear what clinical data are routinely collected. A table summarising these variables, perhaps by meaningful categories, would be helpful for the reader. Please also clarify in the text what data are the core collected data, harmonised across all participating sites, compared to any additional site-specific sources, e.g., the manuscript states the use of additional questionnaires or device data without providing any additional details which I feel are needed for context. Please state why this difference exists and whether there are any plans to expand the use of additional collection forms in all sites.

In addition, do routinely collected data include respiratory assessments, cognitive assessments, anthropometry/body composition data or any information related to significant clinical milestones (for example, non-invasive ventilation initiation or gastrostomy placement)?

Patient and public involvement and engagement is crucial for any patient-facing research activities and I enjoyed reading about the strong involvement of patient feedback in the Consortium. I would recommend perhaps adding a few specific details or examples pertinent to how PPIE has shaped the data collection process (as supplementary material if it cannot fit in the main text), as this might be useful to other organisations planning similar database setups.

The manuscript states that there are specific standard operating procedures for data collection and biosample handling and processing. I wonder whether these are publicly available and if so, a link to this resource would be helpful to readers.

Participants are approached after diagnosis. Is there a specified window for recruitment following diagnosis?

In the findings to date, the cohort amounts to a significant number of people who have been recruited. Do the authors have data on how many potential participants were approached

and how many declined participation? Also, are there any data to indicate why people may decline participation, so does the database miss out on people who lack capacity or are severely impaired, for example? How representative is the cohort sample compared to a typical ALS patient in Australia?

It would be useful for the authors to comment on the extent of completeness/missing data across key clinical variables and biological samples. How consistent is data collection across longitudinal time points? If the dataset is not complete how might missing data influence future analysis?

Collecting longitudinal data in ALS can be particularly challenging and I wonder whether the authors would consider commenting on the operational and logistical challenges they have encountered in their prospective sample and clinical data collection. A mention to specific strategies employed to maximise recruitment and maintain participants for longitudinal data collection would be beneficial for readers.

The medians should be reported with the IQR and a useful addition would be to report the median (IQR) for the time elapsed between symptom onset (not just diagnosis) and the first study visit/data collection appointment.

Reviewer 2

Name Vale, Sancha

Affiliation Federal University of Rio Grande do Norte, Department of Nutrition

Date 11-Nov-2025

COI None

This is an Australian study cohort dedicated to Amyotrophic Lateral Sclerosis (ALS) and Motor Neuron Diseases (MND). This manuscript describes the creation of the consortium, which brings together major MND clinics in Australia to collect longitudinal clinical data and biological samples from patients, controls, and asymptomatic carriers of genetic variants. The main objectives of SALSA-SGC are to build a sustainable research infrastructure, support clinical trials, and facilitate the identification of genomic risk factors and biomarkers, with over 1,800 participants recruited by the end of 2024. The research emphasizes data sharing and collaboration to advance the understanding of ALS.

Comments:

● The supplementary reporting is comprehensive in terms of consent forms and authorship guidelines, and funding is detailed. However, given the observational, cohort profile nature of the study, it is recommended that the authors include and complete the STROBE checklist (Strengthening the Reporting of Observational studies in Epidemiology) as supplementary material to ensure transparency in reporting the cohort establishment methodology.

● The manuscript refers to SALSA-SGC as an "Australian-wide representative cohort", yet explicitly states that the main limitation is that it "only recruits from participating ALS centres [...] and is not a national registry of people with ALS". The authors should qualify the claim of "representativeness" in the Introduction or, ideally, include a section in the Discussion comparing the demographic representativeness (e.g., age, sex, state distribution) of the 1,386 cases against available Australian national ALS/MND population statistics.

● The text mentions that "Some sites administer" the Lifestyle Questionnaire and that longitudinal tracking is available for a subset. The authors should provide a concise table or textual summary quantifying how many participants (or percentage) completed this questionnaire and perhaps summarize the distribution of responses for a few critical environmental or lifestyle variables (e.g., substance use, occupational exposure, or pesticide exposure).

● Authors should include a baseline characteristics table that succinctly presents the distribution of clinical classifications for the 1,333 cases with clinical data. This table should specifically detail the proportion of cases that meet the derived criteria mentioned in the methodology (e.g., the revised El Escorial, Gold Coast, and King stagings) and include a brief discussion explaining the characteristics and implications of the "insufficient information" category.

● The authors could elaborate on how the flexible governance model and the centralized data structure ensure the continuity and availability of the biobank and data for future research after the current cycle of substantial grant funding is exhausted.

● Despite the superior visual impact of figures over tables, the number of figures included in the article is excessive. They have been grouped together, making it difficult to visualize the crucial information that the authors are trying to convey. Simple descriptive statistics that show counts or percentages should be moved to concise tables.

● Clarify the protocol in the "Biological Collections" section. Confirm that 30 mL is the standard volume and state why this volume is necessary. Explain what specific types of tests or products are realized from the 30 mL sample.

● Review the "Participant Eligibility" section for any formatting issues. Checking for improper use of endpoints and consistency in reference style.

VERSION 1 - AUTHOR RESPONSE

Reviewer: 1
Dr. Haris Stavroulakis, Sheffield Institute for Translational Neuroscience

Comments to the Author:
The manuscript describes the significant initiative establishing the Strategic ALS Australia Systems Genomics Consortium (SALSA-SGC). This initiative takes the form of a national prospective cohort study linking rich, longitudinal clinical data with biomarker and 'omics data (including genetic, plasma, and serum samples) across multiple ALS clinics in five

Australian states. While this is not a national register for ALS, the database is unique in matching biological samples to clinical data for 1,386 people living with ALS, plus 388 healthy controls, and 39 asymptomatic carriers, and ALS mimics.

The manuscript is concise and very well written, successfully highlighting the SALSA-SGC dataset as a high-value resource underpinned by a much needed potential to accelerate discovery research and clinical trials in the field of ALS. The number of contributing sites, the robust and well-thought out governance framework, and crucially the longitudinal data collection element are particular strengths that guarantee the long-term scientific value of the dataset and warrant publication.

Having reviewed the manuscript here are some minor comments/suggestions that in my view would improve clarity and strengthen the manuscript further, for the authors to consider.

In the introduction it may be useful to extrapolate from the reported prevalence of ALS in Australia to estimate the total number of people living with ALS at any given time for added context and impact.
I feel a reference or threshold that classifies ALS as a "common" disease based on the approximately 0.3% lifetime risk is required. Otherwise, I would suggest perhaps rephrasing to ALS being "not infrequent" or a similar phrase.

We have changed this sentence
"ALS can be considered a common disease and there remains an urgent need to design more effective treatments"
To
"ALS should not be considered a rare disease and there remains an urgent need to design more effective treatments."

In the cohort description, it is not immediately clear what clinical data are routinely collected. A table summarising these variables, perhaps by meaningful categories, would be helpful for the reader. Please also clarify in the text what data are the core collected data, harmonised across all participating sites, compared to any additional site-specific sources, e.g., the manuscript states the use of additional questionnaires or device data without providing any additional details which I feel are needed for context. Please state why this difference exists and whether there are any plans to expand the use of additional collection forms in all sites.

Thank you for this comment. We now provide a Supplementary Table 2, which gives all clinical fields and their completeness. We have also added this line
Given that data are collected in real-clinic settings complete data cannot always achieved; the numbers presented in the Figures quantify this.
We have also added Supplementary Tables 3 & 4 (see below) and provide more information about differences between clinics (see below).

In addition, do routinely collected data include respiratory assessments, cognitive assessments, anthropometry/body composition data or any information related to significant clinical milestones (for example, non-invasive ventilation initiation or gastrostomy placement)?

Thank you for suggesting this. We have added this text to the end of the clinical data section.
The approved study protocol did not routinely include collection of data associated with respiratory function, cognitive assessments and other clinical information generated as part of clinical care. However, all participants were consented for data sharing of clinical data

generated as part of their care and disease management plans. These were uploaded as reports to the centralised MND research portal so they could be accessed and / or utilised in downstream analyses. Not all participating centres had access to undertake these additional clinical tests and so was not included in the core protocol of the SALSA-SGC.

Patient and public involvement and engagement is crucial for any patient-facing research activities and I enjoyed reading about the strong involvement of patient feedback in the Consortium. I would recommend perhaps adding a few specific details or examples pertinent to how PPIE has shaped the data collection process (as supplementary material if it cannot fit in the main text), as this might be useful to other organisations planning similar database setups.

We agree Patient and Public Involvement and Engagement is important and the community are strongly motivated. We have added this sentence to the PPIE section.
Questions specifically around access and support for care management plans were provided by MND Australia, allowing us to make comparisons across states.

The worldwide ALS community is quite interconnected. We believe anyone setting up a similar database would reach out to us for advice which can be communicated with nuance.

The manuscript states that there are specific standard operating procedures for data collection and biosample handling and processing. I wonder whether these are publicly available and if so, a link to this resource would be helpful to readers.
The last line of the manuscript now reads:
Access to standard operating procedures, laboratory protocols and analysis scripts are available on request.
In this way we can always share the most up to date SOPs.

Participants are approached after diagnosis. Is there a specified window for recruitment following diagnosis?
We have added these sentences to the recruitment paragraph.
These clinics are a mixture of primary and tertiary referral centres. Patients presenting at a primary neurology clinic usually require a diagnostic work-up and are, on average, approached within 3 months of a formal diagnosis. Participants presenting at tertiary clinics have been referred there after diagnosis and so are approached immediately for recruitment. The consulting neurologist decides if a patient is physically and psychologically able to participate.

In the findings to date, the cohort amounts to a significant number of people who have been recruited. Do the authors have data on how many potential participants were approached and how many declined participation? Also, are there any data to indicate why people may decline participation, so does the database miss out on people who lack capacity or are severely impaired, for example? How representative is the cohort sample compared to a typical ALS patient in Australia?
In addition to the text added to the Recruitment Method section, we have added this text to the Cohort characteristics section:
Given data are collected in real-clinic settings complete data cannot always achieved; the numbers presented in the Figures quantify this. Clinics vary in their ability to follow the complete SALSA-SGC protocol; research clinics were more able to collect longitudinal data. Of those invited to participate it is estimated that the vast majority (90%) provide consent. The most common reason to decline is for patients who live in regional and remote parts of the country and only attend a specialised ALS clinic for the purposes of a diagnosis. In these instances, follow-up care and management is undertaken via telehealth and local health practitioners not involved in the SALSA-SGC. Hence, SALSA-SGC cannot be considered a complete registry of ALS in Australia.

It would be useful for the authors to comment on the extent of completeness/missing data across key clinical variables and biological samples. How consistent is data collection across longitudinal time points? If the dataset is not complete how might missing data influence future analysis?
Thank you for this comment, but we are unsure how to provide a response. As with any real clinic data collection, there is missingness and likely missingness not at random.
We believe this line added helps direct the reader to understand the missingness in the data.
Given that data are collected in real-clinic settings complete data cannot always achieved; the numbers presented in the Figures quantify this.

Collecting longitudinal data in ALS can be particularly challenging and I wonder whether the authors would consider commenting on the operational and logistical challenges they have encountered in their prospective sample and clinical data collection. A mention to specific strategies employed to maximise recruitment and maintain participants for longitudinal data collection would be beneficial for readers.
Thank you for this comment. We added this sentence which best explains collection of longitudinal data.
research clinics were more able to allocate time needed, particularly collect longitudinal data

Commented [NW1]: check

The medians should be reported with the IQR and a useful addition would be to report the median (IQR) for the time elapsed between symptom onset (not just diagnosis) and the first study visit/data collection appointment.
We have now added IQR every time we provide a mean or median. We also add
the median sampling delay from disease onset to first biological sample is 20.0 months (IQR 13.0-36.8 months),

Reviewer: 2
Prof. Sancha Vale, Federal University of Rio Grande do Norte, Federal University of Rio Grande do Norte

Comments to the Author:
This is an Australian study cohort dedicated to Amyotrophic Lateral Sclerosis (ALS) and Motor Neuron Diseases (MND). This manuscript describes the creation of the consortium, which brings together major MND clinics in Australia to collect longitudinal clinical data and biological samples from patients, controls, and asymptomatic carriers of genetic variants. The main objectives of SALSA-SGC are to build a sustainable research infrastructure, support clinical trials, and facilitate the identification of genomic risk factors and biomarkers, with over 1,800 participants recruited by the end of 2024. The research emphasizes data sharing and collaboration to advance the understanding of ALS.

Comments:
The supplementary reporting is comprehensive in terms of consent forms and authorship guidelines, and funding is detailed. However, given the observational, cohort profile nature of the study, it is recommended that the authors include and complete the STROBE checklist (Strengthening the Reporting of Observational studies in Epidemiology) as supplementary material to ensure transparency in reporting the cohort establishment methodology.
Thank you, this is a useful suggestion and now state at the end of the introduction

Here, we describe the SALSA-SGC cohort and follow the Strengthening the Reporting of Observational Studies in Epidemiology (STROBE) reporting guidelines for cohort studies (**Supplementary Table 1**).

Our manuscript already endorsed the majority of the check points, but we added this text to the limitations section:
Some clinical sites are more able to support data collection for research than others. Missing data are mostly site dependent, and is not a reflection of patient characteristics. Patients living regionally are under-represented (because their attendance at major ALS clinics in metropolitan sites is limited). To our knowledge, missing data are unlikely to be biased by other patient characteristics, except that patients presenting at an advanced disease stage are less likely to be recommended to participate in SALSA-SGC by their consulting neurologist.

The manuscript refers to SALSA-SGC as an "Australian-wide representative cohort", yet explicitly states that the main limitation is that it "only recruits from participating ALS centres [...] and is not a national registry of people with ALS". The authors should qualify the claim of "representativeness" in the Introduction or, ideally, include a section in the Discussion comparing the demographic representativeness (e.g., age, sex, state distribution) of the 1,386 cases against available Australian national ALS/MND population statistics.
Thank you for this comment. Detailed ALS/MND population statistics are not available. The study is indeed Australia-wide. We have added this sentence to the end of the cohort description, which adds to the new lines in the section based on comments from reviewer 1.
Hence, SALSA-SGC cannot be considered a complete registry of ALS in Australia.

We already state in the limitations section:

The main limitation is that SALSA-SGC only recruits from participating ALS centres across Australia and is not a national registry of people with ALS.

The text mentions that "Some sites administer" the Lifestyle Questionnaire and that longitudinal tracking is available for a subset. The authors should provide a concise table or textual summary quantifying how many participants (or percentage) completed this questionnaire and perhaps summarize the distribution of responses for a few critical environmental or lifestyle variables (e.g., substance use, occupational exposure, or pesticide exposure).

We've added a new supplementary table (Supplementary Table 4) which summarises the distribution of participants and includes distribution of responses for key variables.

Authors should include a baseline characteristics table that succinctly presents the distribution of clinical classifications for the 1,333 cases with clinical data. This table should specifically detail the proportion of cases that meet the derived criteria mentioned in the methodology (e.g., the revised El Escorial, Gold Coast, and King stagings) and include a brief discussion explaining the characteristics and implications of the "insufficient information" category.

We have included clinical characteristics and classifications across different diagnostic criteria (revised El-Escorial, Gold Coast, King's staging) in Supplementary Table 3.
Cohort characteristics are described in **Supplementary Table 3**.

The authors could elaborate on how the flexible governance model and the centralized data structure ensure the continuity and availability of the biobank and data for future research after the current cycle of substantial grant funding is exhausted.

The first line of the Future Direction section now says
Future directions for SALSA-SGC include continued data collection under a flexible governance model (**Supplementary Material 3**), contingent on grant funding,…

The material added into Supplementary Material 3 is:

SALSA-SGC Flexible Governance model

The consortium governance model of SALSA-SGC allows for multiple parties across multiple institutions to co-exist as an entity and can accommodate within its Terms of Reference:
- Human Research Ethics Committee Approvals
- Clinical Trial - Human Research Ethics Committee Approvals
- Collaborative Research Agreements
- Research Service Agreements
- Data and Material Transfer agreements
- Commercial Contracts
- Clinical Standard Practices
- Trading activities

While upholding:
1. Informed consent of the participants
2. Ownership of samples and data by contributing institutions
3. Commercial waivers by participants
4. Open-source requirements by funders and publishers
5. IP generated from accessing data and / or samples
6. Appropriate attribution of contributions of members at time of publishing
7. Distribution of income

Despite the superior visual impact of figures over tables, the number of figures included in the article is excessive. They have been grouped together, making it difficult to visualize the crucial information that the authors are trying to convey. Simple descriptive statistics that show counts or percentages should be moved to concise tables.
Thank you for this suggestion, but our preference to keep this visual summary. We also summarise data available in Supplementary Table 2.

Clarify the protocol in the "Biological Collections" section. Confirm that 30 mL is the standard volume and state why this volume is necessary. Explain what specific types of tests or products are realized from the 30 mL sample.

Thank you for this comment. We had already written that from the 30 mL we store plasma, buffy coat and serum and have described data we have generated. The remainder of the sample is available to researchers. We have not updated the text as we feel everything is included.

Review the "Participant Eligibility" section for any formatting issues. Checking for improper use of endpoints and consistency in reference style.
Done. Thank you

VERSION 2 - REVIEW

**Reviewer**	**1**
**Name**	**Stavroulakis, Haris**
**Affiliation**	**Sheffield Institute for Translational Neuroscience**
**Date**	**10-Feb-2026**
**COI**	

The authors have addressed all comments satisfactorily. I have no further comments to add and would recommend acceptance of the revised manuscript for publication.

**Reviewer**	**2**
**Name**	**Vale, Sancha**
**Affiliation**	**Federal University of Rio Grande do Norte, Department of Nutrition**
**Date**	**13-Feb-2026**
**COI**	

Following the author's response and text revisions, I believe the text is complete and relevant to the study area.
